# Shape-Dependent CO_2_ Hydrogenation to Methanol over Cu_2_O Nanocubes Supported on ZnO

**DOI:** 10.1021/jacs.2c11540

**Published:** 2023-01-30

**Authors:** David Kordus, Jelena Jelic, Mauricio Lopez Luna, Núria J. Divins, Janis Timoshenko, See Wee Chee, Clara Rettenmaier, Jutta Kröhnert, Stefanie Kühl, Annette Trunschke, Robert Schlögl, Felix Studt, Beatriz Roldan Cuenya

**Affiliations:** ‡ Department of Interface Science, Fritz-Haber Institute of the Max Planck Society, 14195Berlin, Germany; § Institute of Catalysis Research and Technology, Karlsruher Institute of Technology, 76344Eggenstein-Leopoldshafen, Germany; † Department of Physics, Ruhr University Bochum, 44780Bochum, Germany; $ Department of Inorganic Chemistry, Fritz-Haber Institute of the Max Planck Society, 14195Berlin, Germany; ∥ Institute for Chemical Technology and Polymer Chemistry, Karlsruhe Institute of Technology, 76131Karlsruhe, Germany

## Abstract

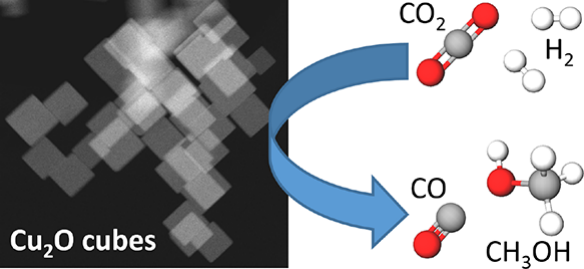

The hydrogenation of CO_2_ to methanol over Cu/ZnO-based catalysts is highly sensitive to the surface composition and catalyst structure. Thus, its optimization requires a deep understanding of the influence of the pre-catalyst structure on its evolution under realistic reaction conditions, including the formation and stabilization of the most active sites. Here, the role of the pre-catalyst shape (cubic vs spherical) in the activity and selectivity of ZnO-supported Cu nanoparticles was investigated during methanol synthesis. A combination of *ex situ*, *in situ*, and *operando* microscopy, spectroscopy, and diffraction methods revealed drastic changes in the morphology and composition of the shaped pre-catalysts under reaction conditions. In particular, the rounding of the cubes and partial loss of the (100) facets were observed, although such motifs remained in smaller domains. Nonetheless, the initial pre-catalyst structure was found to strongly affect its subsequent transformation in the course of the CO_2_ hydrogenation reaction and activity/selectivity trends. In particular, the cubic Cu particles displayed an increased activity for methanol production, although at the cost of a slightly reduced selectivity when compared to similarly sized spherical particles. These findings were rationalized with the help of density functional theory calculations.

## Introduction

The conversion of CO_2_ to methanol through a high pressure hydrogenation reaction is a well-established industrial process.^1,2^ At present, a feed gas mixture that contains H_2_, CO_2_, and CO is used in industry. However, due to the increasing environmental concerns associated with the raising CO_2_ emissions, further efforts to optimize this reaction are highly desirable.

In order to contribute to closing the artificial carbon cycle, CO_2_ should be recycled to produce valuable chemicals.^3−5^ To be independent from the supply of CO, a direct conversion of the greenhouse gas CO_2_ to methanol without CO is preferred. However, a challenge when using a feed gas consisting of CO_2_ and H_2_ to synthesize methanol (eq 1) is the competing reverse water gas shift reaction (RWGS, eq 2), which decreases the selectivity toward methanol by producing CO and water.
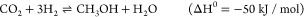
1
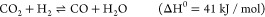
2

To shift the reaction in favor of methanol synthesis, different approaches can be used, including adding promoters,^6−8^ changing the catalyst composition,^9^ or modifying the catalyst’s support material.^10−13^ Another approach consists of exploiting the structure sensitivity of the methanol synthesis^14,15^ and the RWGS reactions.^16^ Previous work on single crystal surfaces showed differences in the CO_2_ hydrogenation to methanol performance when different crystal facet orientations were considered.^17−19^ These works employed bare Cu single crystals,^20−23^ as well as Zn or ZnO-decorated Cu surfaces as model material systems.^17,19,24−27^ On bare Cu single crystals, the activity for methanol synthesis was shown to be (110) > (100) > (111).^18,19^ Interestingly, the same activity order was reported for ZnO-covered Cu surfaces, with the activity of ZnO/Cu(100) > ZnO/Cu(111),^19^ but the trend was modified when metallic Zn was considered on the Cu surface,^27^ with Zn/Cu(111) > Zn/Cu(110). Even though bare Cu(100) surfaces are not expected to show the highest activity for methanol production from CO_2_ with respect to other surface orientations as derived from single crystal works,^18^ the RWGS and the hydrogenation reaction are structure-sensitive to a different degree,^23^ which might lead to a modified selectivity for methanol depending on the initial structure of the Cu pre-catalyst.

The structure sensitivity of the methanol synthesis reaction was also previously demonstrated using nanosized catalysts by comparing the conversion of Cu catalysts of different initial nanoparticle (NP) sizes.^28−30^ Furthermore, structure–reactivity correlations were also shown for the water gas shift^16^ or the CO hydrogenation reactions^31^ by investigating shaped nanoparticle (NP) pre-catalysts. However, a clear restructuring of the particle surface under reaction conditions was observed, even though the general shape of the large particles was preserved. Nonetheless, the step going from the model single crystal systems to morphologically well-defined powder NP pre-catalysts with only one facet primarily exposed is still missing for the CO_2_ hydrogenation reaction.

These academic studies are relevant for practical methanol synthesis with pure CO_2_ as the starting feed. There, the conditions are chosen such as to approach the equilibria of methanol synthesis and water gas shift simultaneously.^32^ Moreover, in order to reach the requested total selectivity for carbon conversion, the reaction is carried out with recycle feed. This means that irrespective of the starting conditions, the catalyst on average and under steady state conditions is exposed to the equilibrium mixture of CO_2_, CO, water, and methanol. Under such conditions, significant restructuring^33^ leads to deactivation. In order to make technical catalysts more resistant against deactivation, it is of utmost relevance to qualitatively understand the kinetically controlled transformations causing deactivation. In particular, it is of relevance to comprehend the known restructuring of the ZnO component^33^ and evaluate more in detail the structural stability of the Cu component, keeping in mind that this component alone exhibits structure sensitivity notwithstanding effects of the synergy between Cu and ZnO.^19^

Here, we investigate Cu_2_O cubes (NCs) supported on ZnO with preferentially exposed Cu(100) facets as model pre-catalysts for CO_2_ hydrogenation and compare their catalytic performance to that of a related system, similarly sized but spherical CuO_*x*_ NPs. Our materials have been selected in order to bridge the complexity gap existing between the industrial nanocrystalline Cu/ZnO/Al_2_O_3_ catalysts and idealized model single crystal or thin films previously employed as proxy for the commercial methanol synthesis catalyst. While our pre-catalyst materials are still structurally (size and shape-controlled) and chemically well-defined, they reflect much better the complexity of the real industrial catalysts, exposing multiple active sites, being amenable for characterization with ensemble averaging-methods, and being highly active under realistic working conditions. In particular, the use of shaped nanoparticle catalysts allows us to unveil the effect of specific surface facets on chemical reactivity without oversimplifying the complexity to that of a much less active single crystal surface. As will be shown below, the present study will allow us to link activity and product selectivity trends to the shape of the initial Cu pre-catalyst, demonstrating that the as-synthesized structure, even though highly dynamic under reaction conditions, still determines the system’s evolution and ultimately steady-state catalytic performance. The insights extracted from this work are expected to contribute to the structural optimization of methanol synthesis catalysts.

### Experimental Section

### Catalyst Preparation

The synthesis of about 70 nm-large Cu_2_O nanocubes (NCs) was based on a previous procedure reported in refs (34, 35). For the synthesis, solutions of 10 mL of CuCl_2_ (0.1 M), 20 mL of l-ascorbic acid (0.1 M), and 30 mL of NaOH (0.2 M) were prepared. First, the CuCl_2_ solution was added into 400 mL of water under continuous stirring, and then NaOH was added. Five minutes after the addition of the NaOH, ascorbic acid was supplied and the solution was stirred for an additional 15 min. During this time, the color of the solution changed from light blue to green and finally to orange. Afterward, the solution was centrifuged and washed with water and ethanol. The final product was stored in pure ethanol. Each batch of the synthesized nanocubes was checked with X-ray photoelectron spectroscopy (XPS) to verify the lack of contamination and with scanning electron microscopy (SEM) and/or scanning transmission electron microscopy (STEM) to monitor the uniformity of the particle size and shape. For the described synthesis, an average cube size of 69 ± 9 nm (Figure S1) was obtained. The final catalyst containing the supported cubes was prepared by drying the solution with the cubes and subsequently mechanically mixing it with commercial ZnO (80–200 nm, US Research nanomaterials Inc.).

A second catalyst with non-cubic particles was prepared with commercial Cu NPs (Sigma-Aldrich) and mixed with the same nanocrystalline ZnO powder. STEM analysis revealed a size of 76 ± 21 nm, which is similar to that of our Cu_2_O cubes. As a second reference, a commercial reference (CR) copper-based methanol synthesis catalyst (45776, Alfa Aesar) was used.

The nominal Cu/Zn atomic ratio selected for the synthesized catalysts was 30/70, except for the commercial reference (63.5% CuO, 25% ZnO, 10% Al_2_O_3_, and 1.5% MgO by weight). The actual ratio in the synthesized catalysts was determined by inductively coupled plasma mass spectroscopy (ICP–MS). For the ICP measurements, 4 mg of catalyst was dissolved in 10 mL of a 1:1:3 mixture of H_2_SO_4_, HNO_3_, and HCl. This solution was digested in a microwave (Anton Paar GmbH, Multiwave GO) at 180 °C for 30 min and diluted with water for the ICP measurements. The results can be found in Table S1.

Specific Cu surface areas were obtained by N_2_O reactive frontal chromatography^36,37^ (N_2_O-RFC). For this, the samples were placed in a fixed-bed reactor and reduced at 250 °C for 2 h in 10% H_2_ prior to the measurement. After this pre-treatment, the catalyst is flushed with He and cooled down to room temperature. Then, a 1% N_2_O in He mixture with a flow of 10 mL/min is introduced and the gas composition at the outlet is recorded with a mass spectrometer. The surface areas are calculated from the difference of the onset for N_2_ (mass 28) and N_2_O (mass 44). The results can be found in Table S2.

### Catalyst Characterization

The size and shape of the bare Cu nanocubes and ZnO-supported cubic Cu catalysts were evaluated by scanning transmission electron microscopy (STEM). STEM analysis was performed with a FEI Talos F200X microscope. The dried powder catalysts were supported on Au lacey carbon grids for these measurements. Additionally, energy-dispersive X-ray analysis (EDX) of the samples was performed to investigate their elemental composition and distribution by collecting elemental maps.

STEM measurements were conducted *ex situ* on the as-prepared samples after air exposure but also on the samples directly after H_2_ reduction and after the CO_2_ + H_2_ reaction without air exposure, i.e., preserving and transferring them in an inert atmosphere. After taking the catalyst out of the reactor, a dark red color is observed, which suggests the presence of reduced copper. The color changes from red to black within a few minutes when exposed to air because of the oxidation of metallic Cu to CuO. To avoid this change of the chemical state and possibly also of the structure of the catalyst, the reactor tube containing the catalytic bed was sealed in our flow reactor and directly transferred to an Ar-filled glovebox. Inside the glovebox, the TEM grid was prepared and mounted on a vacuum transfer TEM holder that was sealed against the outer atmosphere. The TEM holder was only opened after it was placed inside the already evacuated TEM. This way, the exposure of the sample to oxygen is avoided.

The chemical surface composition of the samples was evaluated by X-ray photoelectron spectroscopy (XPS) with a monochromatic Al Kα X-ray source (*E*_Kα_ = 1486.7 eV) and hemispherical analyzer (Phoibos 150, SPECS GmbH) under ultrahigh vacuum (UHV, <10^–9^ mbar) conditions. A flood gun was used to compensate for sample charging.

*Operando* X-ray absorption near-edge structure (XANES) and extended X-ray absorption fine-structure (EXAFS) data were acquired at beamline 2-2 of SSRL (Stanford Synchrotron Radiation Lightsource). All measurements were done in transmission mode. For the measurements, the catalyst was loaded into a quartz capillary that was connected to a gas dosing system and a mass spectrometer for product analysis. XAS data alignment, normalization, and linear combination fitting were done with the Athena software.^38^ The catalysts were diluted by a factor of 1:7 with SiO_2_ for optimal signal quality in transmission configuration.

X-ray diffraction (XRD) patterns were recorded using a Bruker AXS D8 Advance diffractometer equipped with a reaction cell for *in situ* measurements of the catalysts. A Cu Kα source and a position-sensitive energy-dispersive detector (LynxEye XE-T) were used for the experiments. XRD patterns were recorded in continuous scanning mode in a 2θ range of 28–85° applying an increment of 0.005°. XRD analysis was performed using the EVA/TOPAS software package (Bruker).

Diffuse reflectance infrared Fourier transform spectroscopy (DRIFTS) measurements were carried out with a Cary 680 FTIR spectrometer (Agilent) equipped with a diffuse reflectance accessory and low temperature reaction chamber (Harrick Praying Mantis DRP-DF8 and chamber CHC-CHA-3). A nitrogen-cooled MCT detector was used for the collection of the spectra with a spectral resolution of 2 cm^–1^. The spectra shown are the accumulation of either 512 or 4096 (for high quality spectra) consecutive scans. Prior to the experiments, the samples were reduced in 10% H_2_ in He at 250 °C for 30 min. Afterwards, the chamber was first flushed with pure He, then evacuated to high vacuum (10^–5^ to 10^–6^ mbar), and finally cooled down to liquid nitrogen temperatures. For CO adsorption measurements, CO was dosed stepwise at increasing pressures from 0.5 to 500 mbar, recording a spectrum at every step. Afterward, the reaction cell was again evacuated stepwise down to high vacuum to observe the desorption behavior of CO on the catalysts.

*In situ* Raman spectroscopy was measured with a confocal Raman spectrometer (Renishaw, inVia Reflex) combined with a home-built reaction cell attached to a motorized stage for sample tracking (Renishaw, MS300 encoded stage). The spectrometer was calibrated using a Si (100) wafer (520.5 cm^–1^) before each experiment. The spectra were acquired using a near-infrared laser (λ = 785 nm, *P*_max_ = 500 mW) and a green laser (λ = 532 nm, *P*_max_ = 500 mW). The spectrometer was coupled with a microscope (Leica Microsystems, DM2500M) equipped with a super-long working distance objective (Nikon TU Plan EPI ELWD, 50×). The reaction cell is a stainless steel tube with a 1 mm wide slit covered with a sapphire window. The cell is able to achieve pressures up to 15 bar and a temperature of at least 250 °C. The sample was first measured in its initial state in air. Afterwards, the cell was flushed with He and the sample was reduced in an atmosphere containing 10% H_2_ in He at 250 °C. In the last step, the reaction mixture (60% H_2_ + 20% CO_2_ + 20% He) was introduced and the pressure was increased to 15 bar. Raman spectra were recorded at multiple temperatures in subsequent steps under reaction conditions, namely, 250, 220, 170 °C, and room temperature.

### Catalytic Activity

The catalytic performance was tested in a fixed-bed mass flow reactor. 100 mg of catalyst was diluted with SiC (1:6 by mass) and then placed in a glass-lined steel reactor tube. Before testing, all catalysts were reduced under a flow of 10% H_2_ in He at 250 °C for 2 h. The activity was measured at pressures of 20, 40, and 60 bar and temperatures of 220, 250, and 280 °C. Additional runs were done at 40 bar and 170 °C in the reaction gas mixture. The reaction gas mixture consisted of CO_2_, H_2_, and He (20:60:20), with the latter being used as the internal standard. The total flow during the reaction was 50 mL/min. The reaction products were measured by online gas chromatography (GC) with an Agilent Technologies 7890B GC equipped with a flame ionization (FID) and two thermal conductivity detectors (TCD). All values given were obtained after a steady-state activity was reached. The data presented are the average of at least three consecutive injections, unless specifically noted. The time between injections was 20 min.

### Theory

Density functional theory calculations were performed using the Vienna ab initio simulation package (VASP)^39,40^ in connection with the atomic simulation environment (ASE).^41^ A plane-wave basis set with a cutoff energy of 450 eV, the projector augmented wave method (PAW),^42,43^ and the Bayesian Error Estimation Functional with a van der Waals (BEEF-vdW)^44^ exchange correlation functional were used. The choice of the BEEF-vdW functional is motivated by its performance regarding adsorption energies and transition states on transition metal surfaces^45−47^ and its description related to CO_2_ hydrogenation to methanol.^48^

Copper infinite slab models consisting of four layer-thick 3 × 3 super cells, separated by more than 15 Å in the *z* direction and one surface row substituted with Zn atoms, were used to model the ZnCu(100) and ZnCu(211) surfaces. The top two layers and the adsorbates were allowed to relax, whereas the bottom two layers were kept fixed during geometry optimization and the convergence criterion was a maximum force of 0.01 eV/Å. The Brillouin zones were sampled using 4 × 4 × 1 and 5 × 4 × 1 Monkhorst–Pack *k*-point grids^49^ for the ZnCu(100) and ZnCu(211) models, respectively. Transition states were obtained using constrained optimizations, and all transition states were verified to contain only one imaginary harmonic frequency corresponding to the transition vector of the reaction. The ZnO/Cu(111) system has been modeled using a four layer-thick 7 × 4 large Cu(111) unit cell with 3 × 3 large single layer ZnO nanowires (truncated in the *x* direction and infinite in the *y* direction). The ZnO/Cu(100) systems have been modeled using a four layer-thick 4 × 6 large Cu(100) unit cell with a 3 × 3 large single layer ZnO nanowire (truncated in the *y* direction and infinite in the *x* direction). The Brillouin zones were sampled using 2 × 4 × 1 and 3 × 2 × 1 Monkhorst–Pack *k*-point grids^49^ for the ZnO/Cu(111) and ZnO/Cu(100) models, respectively (details in the SI).

## Results and Discussion

### Catalyst Structure, Morphology, and Composition: As-Prepared, after Reduction, and during Reaction

**Figure 1:**
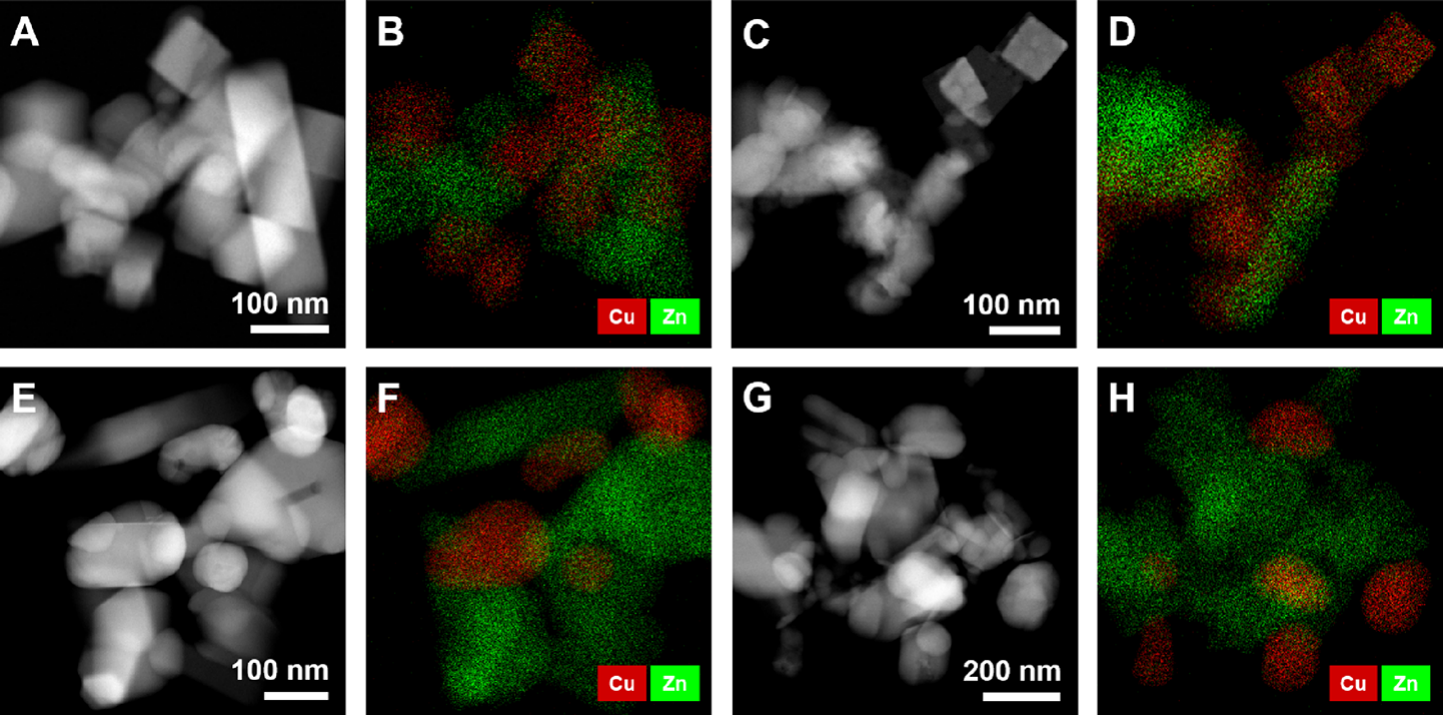
STEM images and corresponding EDX maps of cubic Cu_2_O NPs supported on nanocrystalline ZnO: (A, B) as prepared Cu_2_O, (C, D) after reduction in H_2_ at 170 °C for 2 h, and after the CO_2_ hydrogenation reaction at 170 °C for (E, F) 10 min and (G, H) over 100 h. The green color in the EDX maps corresponds to Zn, the red to Cu.

The cubic morphology of our as-prepared Cu_2_O catalysts was confirmed by high-angle annular dark-field (HAADF)-STEM, shown in Figure 1A, and an average cube edge length of 69 ± 9 nm (Figure S1) was determined. After supporting the Cu_2_O cubes on ZnO, the STEM measurements revealed that the cubic shape was preserved (Figure 1A). The Cu NPs were identified by the EDX mapping. The EDX spectra corresponding to the EDX maps shown in Figure 1 can be found in Figure S2. As seen from the elemental maps, the particles are well mixed with the ZnO support (Figure 1B). However, some changes were already observed in the Cu catalyst morphology upon reduction in hydrogen before reaction. Figure 1C shows the catalyst after exposure to 10% H_2_ (balanced in He) at 170 °C for 2 h. While some particles remain cubic, others start to deform and become more rounded. When performing the reaction under CO_2_ hydrogenation conditions (20% CO_2_ + 60% H_2_ + 20% He, 170 °C, 40 bar) after the H_2_ reduction, more drastic morphological changes are observed, Figure 1E. Even after a short reaction time (10 min), numerous rounded Cu particles are visible, which start sintering when multiple particles are close to each other. Keeping the particles under these conditions for a longer time (>100 h, Figure 1G) results in almost all particles being deformed, and a larger average particle size than that of the initial cubic pre-catalyst, with some NPs >100 nm. The morphology of the ZnO support seems to be less affected by the reaction.

**Figure 2:**
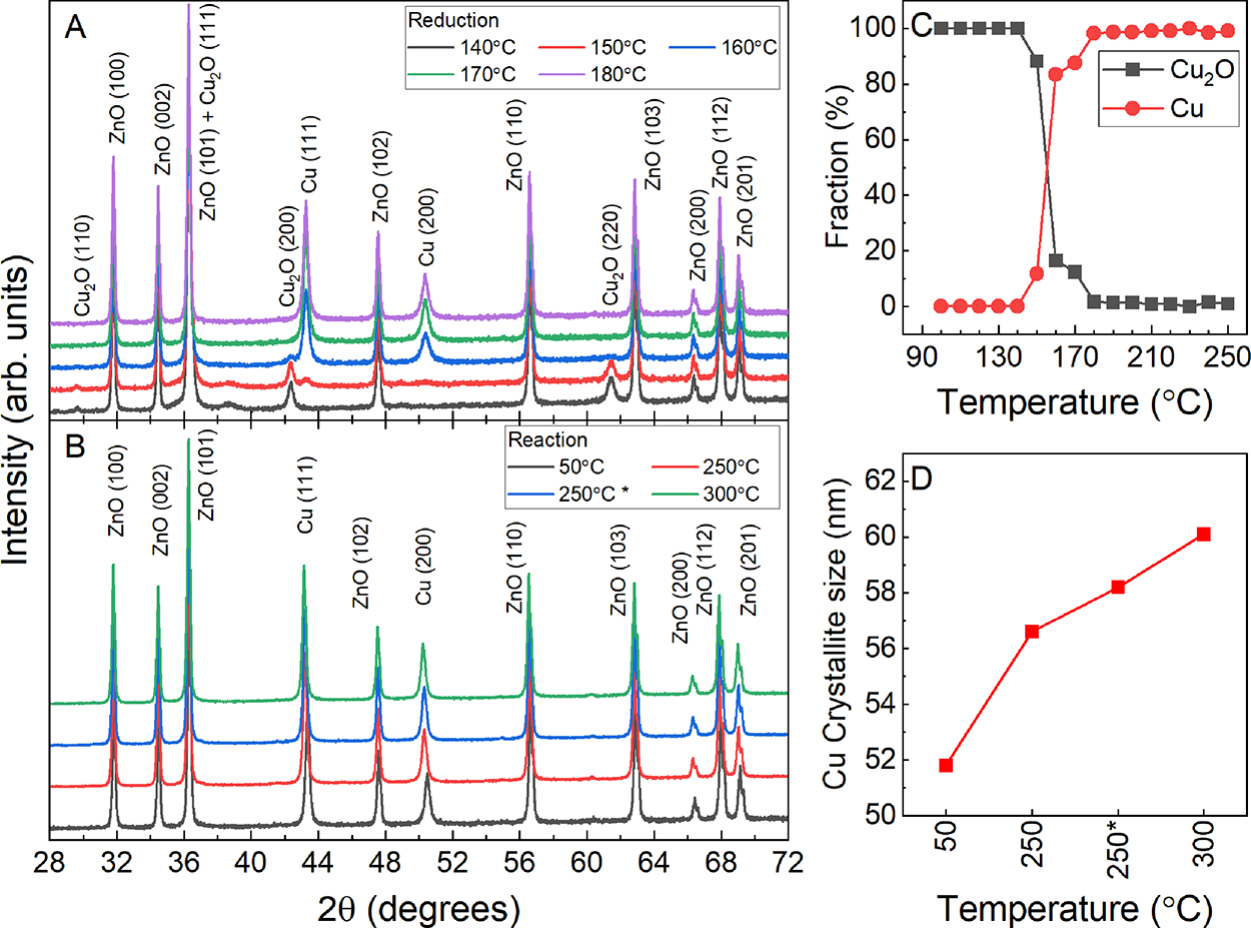
*In situ* XRD measurements of Cu_2_O cubes supported on ZnO. (A) Diffractogram acquired during the reduction with 10% H_2_ balanced in He between 140 and 180 °C and (B) under reaction conditions (75% H_2_ + 25% CO_2_, *p* = 10 bar) at the indicated temperatures. (C) Evolution of the concentration of the oxidized and metallic copper species during the reduction. One step at each of the indicated temperatures lasted approximately 12 min. (D) Cu crystallite size extracted by Rietveld fitting analysis for the catalyst under reaction conditions. During the reduction, scans were started immediately after the desired temperature was reached. All scans in the reaction mixture were started once the desired temperature was reached and was stable for 30 min, except the scan labeled as 250*, which was performed after 2 h in the reaction mixture at 250 °C.

**Figure 3:**
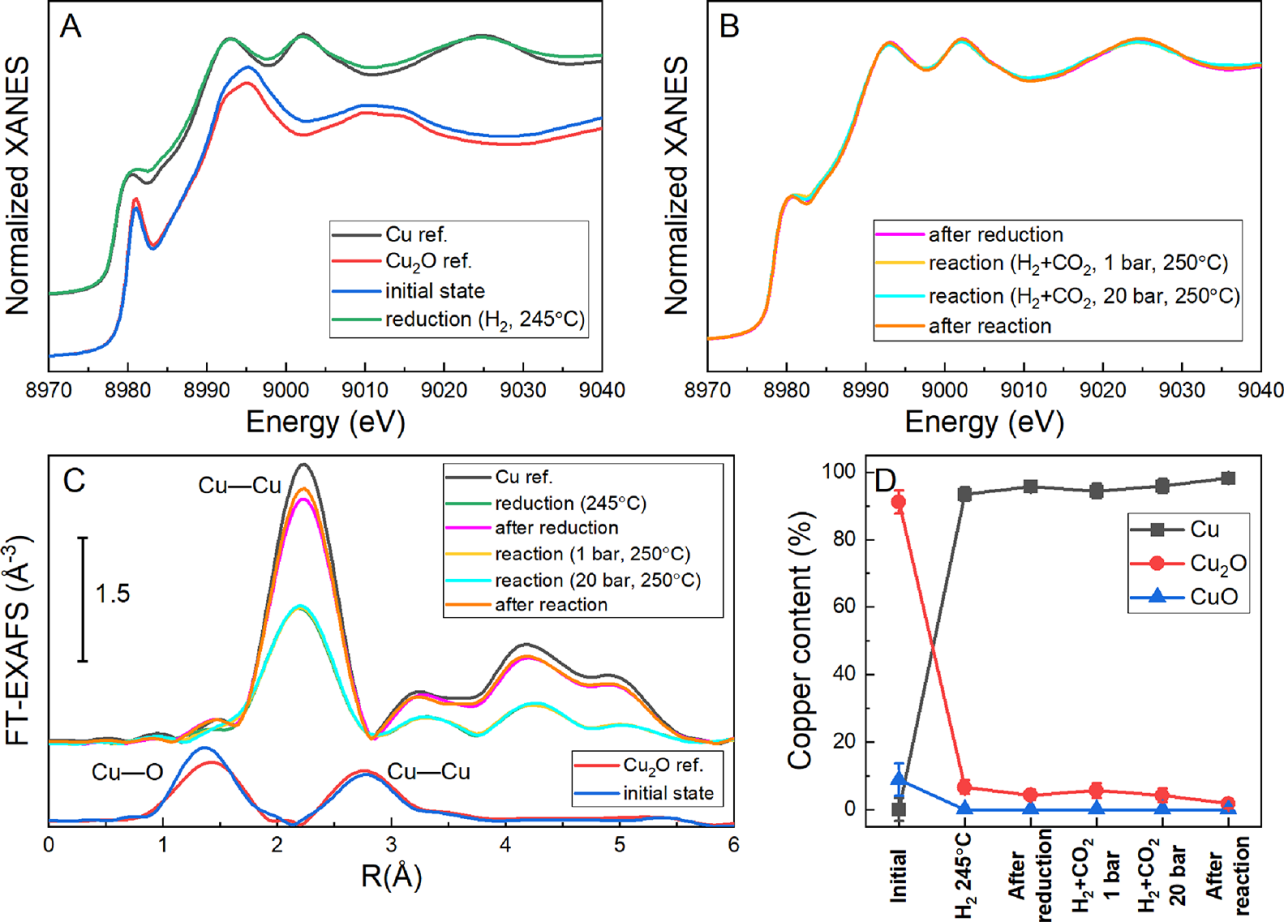
*Operando* (A, B) Cu K-edge XANES and (C) Fourier-transformed EXAFS (*k*-weight = 2) spectra of the NCs on ZnO catalyst during and between the different treatments as indicated inthe figure. Spectra labeled “after reaction/reduction” were acquired in a He atmosphere at room temperature. Additionally, reference spectra of bulk Cu_2_O and Cu are displayed. (D) Results of the linear combination fitting of the Cu K-edge XANES spectra collected during the different pre-treatment and reaction steps.

Spectroscopy (XPS, XAS) and diffraction (XRD) measurements confirmed that the primary phase of the as-synthesized cubes is Cu_2_O (see Figures 2 and 3 and Figure S3). A low Cu^2+^ contribution observed in the XPS spectra suggests the oxidation of the nanocubes’ outer surface layer to CuO (Figure S3) due to the exposure to air prior to the measurement. Fitting of the Cu LMM Auger spectra (Figure S3B) with a linear combination of reference CuO and Cu_2_O spectra provided 15% CuO and 85% Cu_2_O. The presence of CuO was also detected by XAS but at a slightly lower concentration of 8.8% due to the bulk sensitivity of XAS (Figure 3D). No metallic copper was detected in the nanocube catalyst before reduction.

Pretreatment and reaction were investigated by means of *in situ* XRD (Figure 2). The catalyst reduction was performed in 10% H_2_ (balanced in He at atmospheric pressure) in 10 °C steps starting at 100 °C, and the XRD data were collected at each temperature for 12 min. XRD showed the complete reduction of the cubic Cu_2_O pre-catalyst to metallic Cu during the activation treatment in hydrogen, with an onset reduction temperature of 150 °C. At 180 °C, the Cu_2_O component had completely disappeared and only metallic copper was detected by XRD (Figure 2C). The Zn component is present as ZnO in its typical wurtzite structure and remains unchanged during the reduction (Figure 2A). Following the reduction, similar XRD measurements at 10 bar were carried out in the CO_2_ + H_2_ (1:3) reaction mixture (Figure 2B), with scans lasting 40 min. After acquiring XRD data at 50 °C, the sample was heated in the reactant mixture to 250 °C, where two scans were performed after 30 min and after 2 h. Subsequently, an additional scan was performed at 300 °C, staying 30 min at this temperature. Overall, no notable changes are observed by XRD regarding the oxidation state of Cu under reaction conditions (CO_2_ + H_2_), which remains metallic. However, Rietveld analysis showed a slight increase in the Cu crystallite size from 52 to 60 nm over time at elevated temperatures (≥250 °C), Figure 2D, hinting to the onset of Cu NP sintering over time. No changes of the ZnO support could be detected with XRD under reaction conditions. For both, the Cu and ZnO peaks, a shift is observed in the peak position as a function of the applied temperature. By comparison to reference samples, this can be completely attributed to a thermal effect.

A sample consisting of spherical Cu NPs supported on ZnO was also investigated by *in situ* XRD (Figure S4). This sample started with a higher initial fraction of metallic Cu (65%), but displayed the same behavior as the Cu_2_O NCs, with an onset temperature for the Cu oxide reduction of 150 °C. The crystallite size of this sample extracted by Rietveld analysis is similar to the cubic particles, which was expected since both types of particles were initially similarly sized. Furthermore, just like the cubic particles, the crystallite size of the spherical NPs also increases when exposed to higher temperatures (≥250 °C). In summary, both samples, the cubic and the spherical Cu NPs supported on ZnO, show the same structural and chemical evolution in XRD.

Additional information on the evolution of the structure and composition of the cubic nanocatalysts was extracted from *operando* XAS measurements (Figure 3, Figures S5 and S6). The progressive Cu_2_O reduction was monitored by XANES in 20% H_2_ (balanced in He) up to 245 °C, with a heating rate of 10 °C/min. In agreement with the XRD findings, a Cu K-edge XANES scan performed at 150 °C during the reduction showed the first slight changes, hinting that the onset of the Cu_2_O reduction is at this temperature. Already at 200 °C, almost complete reduction of Cu was observed. This is consistent with the XRD measurements. A linear combination fit of the XANES spectra using the spectra for a Cu foil, Cu_2_O, and CuO as references showed that even after the reduction treatment, a small fraction of Cu is present as Cu_2_O. This small contribution (1–7%) remains throughout the experiment (Figure 3D). It should be noted however that the content of these minority species is close to the uncertainty of the linear combination fit. The sample was cooled to room temperature (RT) after the reaction test and high-quality EXAFS spectra were measured in He.

Subsequently, the reaction gas mixture (75% H_2_ + 25% CO_2_) was introduced and the sample was heated up to 250 °C and measured at 1 and 20 bar. Finally, EXAFS spectra for the sample were collected again at RT in He. Interestingly, once the reduction treatment is completed, the XANES and EXAFS signals remain unchanged for both, the Cu K-edge and the Zn K-edge. In fact, the ZnO component (Figure S6) hardly changes over the course of the whole experiment. The variations in the Cu K-edge EXAFS signal shown in Figure 3C and Figure S5 are largely due to the different temperatures of the measurements, and thus different thermal disorder induced in the sample.

Close inspection of the data in Figure 3 and Figure S5 reveal that despite the fact that the overwhelming majority of the Cu atoms can be found in the perfect order of a metallic lattice, there is still a tiny fraction of deviating species. Linear combination analysis shows an amount of about 2% Cu_2_O, even after the completion of the experiment, which includes the initial reduction procedure and the treatment under reaction conditions (20 bar and 250 °C). This points to some disorder of unspecified origin which is, however, an important indication when explaining the origin of the morphological transformations described above. The Cu metal obtained by the present synthesis procedure is not in its equilibrium form but contains defective minority species possibly connected with residual oxygen. Similar observations made with other spectroscopic probes are described below.

### Catalytic Performance

**Figure 4:**
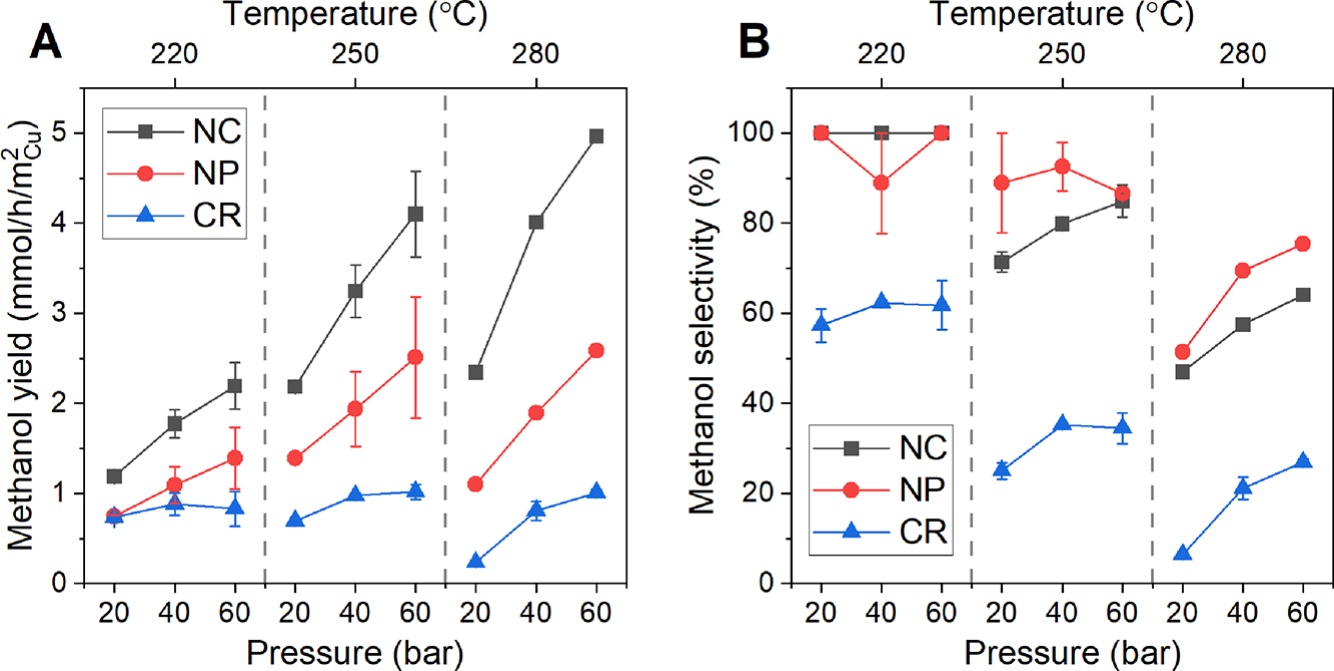
(A) Methanol yield and (B) selectivity of Cu_2_O nanocubes (NC) on ZnO, spherical Cu NPs on ZnO (NP), and the commercial reference catalyst (CR). The lines in the plots are just guides for the eye. All experiments were done with one sample of each catalyst used subsequently for each parameter variation.

In the following, we compare the catalytic performance of the three investigated catalysts, which are pre-shaped Cu_2_O nanocubes on ZnO (NCs), spherical Cu nanoparticles on ZnO (NPs), and a commercial reference catalyst (CR). Figure 4 displays the methanol yield and product selectivity as a function of the reaction (CO_2_ + H_2_) pressure and temperature after a stable production was reached. Generally, the catalytic activity was found to increase with increasing temperature and pressure for all catalysts. The methanol yield was indeed higher with increasing pressure (Figure 4A), but no significant increase was observed for the highest measurement temperature. In fact, for the commercial reference, a decrease in the methanol yield was observed at 280 °C due to the lower selectivity toward methanol under these conditions (Figure 4B). Instead, the CO production due to the RWGS reaction was found to increase. Note that the methanol yield in Figure 4 is normalized by the Cu surface obtained from N_2_O-RFC measurements (Table S2). Interestingly, regardless of the original particle shape (cubic vs spherical), both of the ∼70 nm Cu/ZnO catalysts (NPs and NCs) were superior to the commercial reference with respect to the methanol yield when normalized by the Cu surface area. The deviation between the commercial reference and the other catalysts originates from the different structures and compositions. A commercial catalyst is optimized for maximum methanol yield per weight of catalyst and is usually operated including CO in the feed gas composition.^32^ The total amount of methanol produced per gram of catalyst here is indeed higher for the commercial catalyst as shown in Figure S7. In addition, for the latter sample, the methanol production rate was not found to change significantly within the 250 to 280 °C temperature range, in contrast to the observation made for the other two ∼70 nm cubic and spherical Cu/ZnO catalysts. This observation is assigned to the increased selectivity toward CO at higher temperatures due to the competing RWGS reaction.

Apart from methanol and CO, only trace amounts of methane were detected (<0.2%) as a byproduct for all catalysts. The best methanol/CO ratios were obtained for all catalysts at the highest pressures and at the lowest temperatures.

When comparing the catalytic performance of the non-cubic NPs to that of the nanocubes on ZnO, both catalysts behaved very similarly, but the NC catalyst had clearly an overall higher methanol production and only slightly lower methanol selectivity. We assume that, despite the initial shape transformations, a higher fraction of the surface of the nanocubes will still have the Cu(100) facet preferentially exposed. The non-preshaped particles in contrast will most likely have the thermodynamically more stable Cu(111) facet exposed, mixed with Cu(211) steps responsible for part of the methanol activity. This is in line with previous single crystal studies^18,19^ that revealed a higher methanol yield on Cu(100) as compared to Cu(111).

The conversion tests were done such that for the model systems, the performance was well within the kinetic regime with no influence of equilibration. The reference catalyst is initially about three times more productive than the model systems and undergoes transformation to brass after the first high-temperature episode. This explains the diverging trend in performance seen in Figure 4. It is remarkable that either the number density or the quality of the active sites seem superior in the model systems as compared to the reference catalyst. This could be due to the normalization against the N_2_O surface area. If there prevails a structure sensitivity effect embedded in the reactivity of methanol synthesis catalysts, as implied by the present observations, this would open up a new avenue to further boost the performance of this critical catalytic transformation.^50^

**Figure 5:**
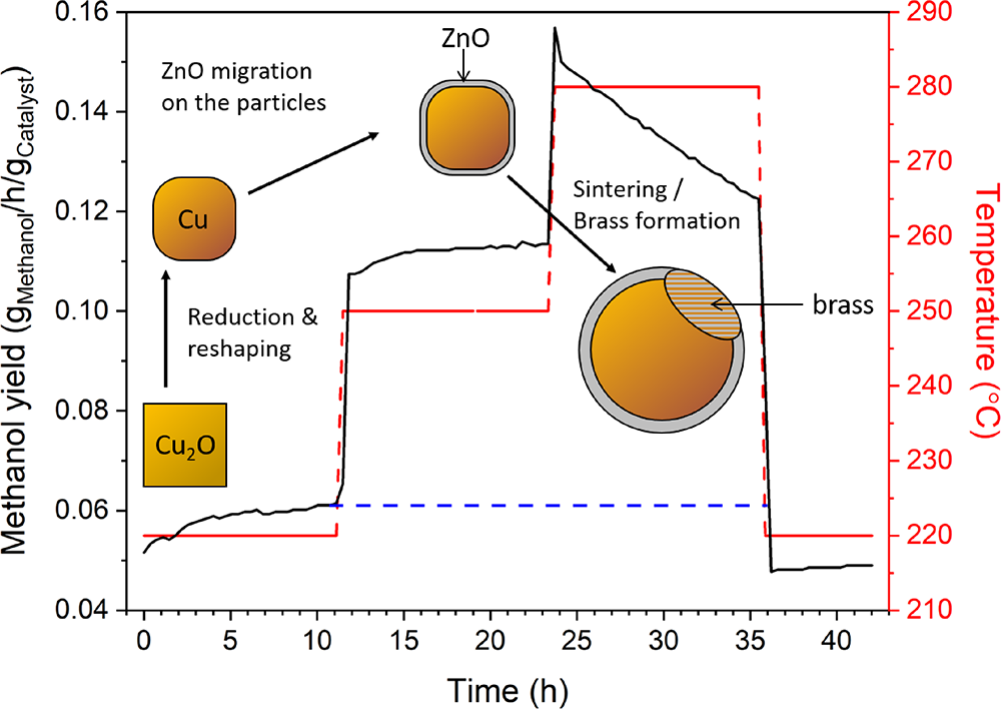
Methanol production (normalized by the weight of the catalyst) of the Cu_2_O NC catalyst at 20 bar acquired at different temperatures. The dashed blue line serves as a guide for the eye to compare activity at 220 °C before and after the 280 °C reaction step. The inserted drawings illustrate the changes happening to the NC catalyst during the reduction treatment and also in the course of the reaction, including the formation of a ZnO overlayer and the sintering and possible brass formation at high temperature leading to the deactivation.

We also explored the temporal evolution and deactivation of the cubic pre-catalysts. The observed change of the shape of the Cu NCs (rounding) and size (increase from 69 nm to 100–200 nm) with extended time under reaction conditions (150 h) is accompanied by a change of the catalytic activity. These changes are also tied to the selected reaction temperature. Figure 5 shows the temporal evolution of the methanol production at various temperatures for the cubic particles. At the beginning of the experiment at 220 °C and 20 bar, the catalytic activity increases until it reaches a stable steady-state value. This value remains stable at reaction temperatures of 220 and 250 °C, but at 280 °C, the catalytic activity starts to decrease. This can be mainly explained by sintering of the Cu particles, which is known to be the main source for deactivation of Cu/ZnO catalysts.^51,52^ This is further evidenced by the increasing crystallite size observed in our XRD measurements during the reaction and also in the TEM images acquired after the reaction. Also, the formation of a Cu-Zn alloy would lead to a worse catalytic performance.^13,53^ Nonetheless, drastic brass formation has been typically reported at higher temperatures than the ones applied here^54^ or in more reducing gas mixtures^55^ (for example, when CO is added or when the CO_2_/H_2_ ratio is reduced). Moreover, even if alloying occurs, the stability of brass species under reaction conditions has been described to be limited in a H_2_ + CO_2_ feed at the applied temperatures.^56^ However, the reduction of Zn and with it, the formation of a Cu-Zn alloy, is heavily dependent on multiple parameters, such as the surrounding atmosphere, temperature, or even the Zn coverage on Cu. Therefore, we cannot completely rule out the formation of small amounts of brass in this study and it is also clear that the deactivation observed is much more severe at 280 °C, when brass is expected to be more easily formed, than at 220 °C or even 250 °C. Nonetheless, our XRD and TEM data clearly indicate that sintering takes place at these high temperatures, which is very likely the main cause of the fast deactivation observed here. This may also be related to the fact that at 280 °C more water is produced. Because we did not add CO to the reactant mix, the excess water is not easily removed by the water gas shift. Water is indeed detrimental to the stability of the catalyst^52,55,57^ and encourages sintering. Furthermore, it has also been proposed that water also disrupts the Cu–ZnO synergy and can destroy active sites by crystallization of ZnO.^33,52^

The deactivation of the catalyst is an irreversible change, which is associated with a decreased methanol production when returning to the initial reaction temperature of 220 °C after the high temperature step (Figure 5). Furthermore, this deactivation behavior is observed for the spherical NPs as well, where sintering of the particles was also observed via TEM after the reaction, Figure 1.

To further investigate structure–reactivity correlations, we performed an additional experiment in CO_2_ + H_2_ at 40 bar and 170 °C. The latter temperature was chosen because it is the lowest temperature where the catalyst is active but also mainly reduced. As one can see in Figure S8, the activity increases again, but this time more slowly over time, since more than 40 h (vs typically 4 h) were required until stable methanol production was reached. Interestingly, no subsequent decrease of the catalytic activity was observed. Furthermore, when flushing the reactor with He and subsequently re-starting the reaction, the former methanol production was achieved immediately. The lack of deactivation here also goes hand and hand with the lack of particle sintering observed at this low temperature. Furthermore, the initial activity increase detected cannot be related to an additional reduction of the Cu component of the catalyst, since we can follow the consumption of H_2_ during the reduction by mass spectrometry (Figure S9) and complete reduction of Cu only takes about 20 min. The reducing potential of the pure H_2_ feed during the activation should also be higher than when adding CO_2_ to the feed, despite being at higher pressure.^56^ Therefore, a slow CuO_*x*_ reduction is unlikely and the activity increase must be related to a different effect. The most plausible explanation is the progressive migration of ZnO species from the support to the Cu particle surface. This behavior was reported before,^33,58^ and the addition of ZnO is well known to be beneficial for the catalytic activity and methanol selectivity.^13^ The migration of Zn species might already happen during the pretreatment of the catalyst, but the formation of the active Cu-ZnO sites may only occur when CO_2_ is added.^59,60^

Furthermore, for a similar initial NP size (∼70 nm), the distinct initial shape of the pre-catalysts (cubic vs spherical) is also expected to influence the Cu–ZnO interaction since different surface structures and support contact areas are expected for the spherical NPs vs the NCs. The segregation of ZnO from the support onto the different Cu surfaces is expected to influence the further evolution of the catalyst. It is plausible that the modified surface of the cubic particles with ZnO consequently also affects their activity and selectivity due to the changed initial Cu–ZnO structure and the resulting electronic modifications. Nonetheless, ultimately this would be an effect attributed to the different Cu facets in the pre-catalyst that are exposed during its activation. The differences resulting in a dissimilar catalytic performance observed between the NCs and spherical NPs are possibly already (partially) established during the activation, with a more facile metal particle–support interaction for the NCs due to their initial larger contact area with the ZnO support.

### Theoretical Calculations

**Figure 6:**
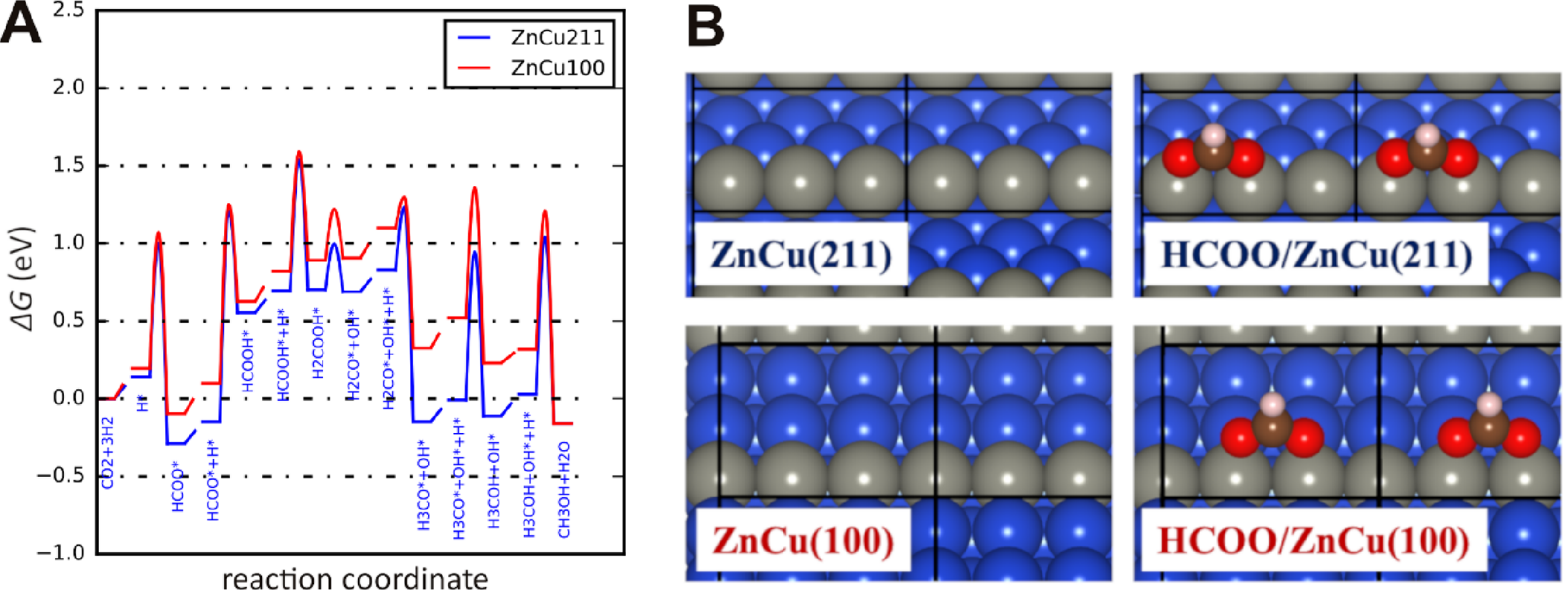
(A) Calculated Gibbs free energy diagram for CO_2_ hydrogenation to methanol (*T* = 227 °C (500 K), *p*(H_2_) = 40 bar, *p*(CO_2_) = 10 bar, and *p*(CH_3_OH) = 1 bar) over Cu(100) (red) and Cu(211) (blue) surfaces. (B) Structures of ZnCu(211) and ZnCu(100) surfaces used in the calculations and optimized structure of adsorbed formate. Blue = Cu, gray = Zn, red = O, brown = C and white = H.

In order to gain further insight into the effects of the NP shape and corresponding surface orientation on the CO_2_ hydrogenation activity to methanol, we turned to density functional theory (DFT) calculations of the related surface processes. Our starting point is the Cu(100) facet that we expect to constitute the majority of surface area of the cubic pre-catalysts under reaction conditions, despite the drastic structural changes previously reported under working conditions. Following models of surface alloy formation,^61^ we use a ZnCu(100) surface where 1/3 ML of the surface copper is substituted by Zn (see Figure 6). We compare these results to stepped ZnCu(211) surfaces, also being ZnCu surface alloys, that have been invoked in earlier studies^15^ and are thought to represent defective NP surfaces. It should be noted that although ZnCu alloys have been experimentally reported to be less active than ZnO/Cu for this reaction,^13,53^ such surfaces still make methanol from CO_2_ + H_2_.

The results are shown as a free energy diagram at 227 °C (500 K), high pressures (40 bar H_2_, 10 bar CO_2_), and low conversion (1 bar methanol) in Figure 6. The overall mechanism and free energy pathways are comparable to those reported in earlier studies using similar computational parameters and the ZnCu(211) surface.^15,62^ Our calculations reveal that the intermediates and transition states involved in CO_2_ hydrogenation are fairly similar for ZnCu(100) when compared to the ZnCu(211) surface. This is particularly the case for the presumably rate-determining reaction step from HCOOH* to H_2_COOH*, where both surfaces have free energy barriers of approximately 1.5 eV when compared to gas-phase CO_2_ and H_2_. The only pronounced difference in the reaction energetics is given by the weaker adsorption of formate (HCOO*) on the ZnCu(100) surface. In fact, the formate intermediate is exothermic on ZnCu(211) (−0.29 eV), whereas it adsorbs with a slightly negative adsorption energy on ZnCu(100) (−0.10 eV). This indicates that while ZnCu(211) will be covered by close to a monolayer of formate, as has been shown through kinetic modeling,^15^ the formate coverage on ZnCu(100) will be less pronounced. Note that this relates to formate binding energies, where formate binds to two surface Zn atoms, both for ZnCu(211) and ZnCu(100).

The observed similar free energy profiles reveal that the activity of ZnCu(100) should be rather high. Given that the majority of nanocubes expose the (100) surface, one would expect a high activity per total copper surface area. On the other hand, ZnCu(211) facets, that might be representative of the active sites of the spherical NPs, only constitute a minor fraction of the overall surface area of those NPs. It should be noted that even though our initial cubic Cu_2_O NPs were found to experience drastic morphological changes during the pre-treatment in H_2_ and subsequent CO_2_ + H_2_ reaction, it is plausible that a preferred (100) orientation remains as smaller domains or facets, which might explain the superior activity of the cubic Cu_2_O pre-catalysts. The spherical NPs would probably have primarily (111) facts exposed since they are the most thermodynamically stable facets.

Nonetheless, it is expected from the literature that the surface of the Cu nanocubes and the spherical Cu NPs is covered by ZnO during the reaction,^13,58^ as will be demonstrated below based on spectroscopy measurements, which would affect the binding of the reaction intermediates.

**Figure 7:**
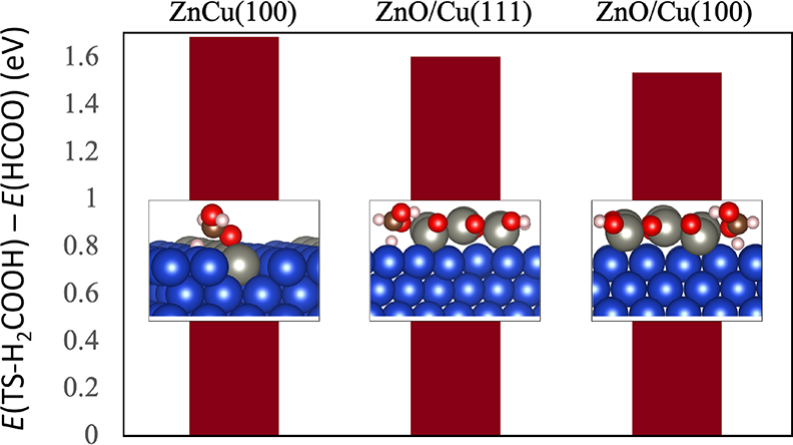
Calculated difference between energies of the transition state of H_2_COOH and adsorbed formate on the surface for ZnCu(100) compared to the employed models for ZnO/Cu(111) and ZnO/Cu(100). Structure of the optimized transition state of H_2_COOH formation for the three surfaces. Color code is the same as in Figure 6.

To study whether ZnO on the copper surfaces can also have an influence on the reaction mechanism, we have also investigated Cu(111) and Cu(100) surfaces with adsorbed ZnO. The exact structure of the ZnO overlayer is hereby unclear and might also evolve under reaction conditions as Zn is mobile on the Cu surface. Therefore, we had to choose a sensible model whose structure is likely to resemble the real state. This model was constructed employing ZnO stripes to mimic the ZnO/Cu interface, as shown in Figure 7. Due to the strong hydrogen binding to the oxygen-terminated ZnO (see the SI), those oxygen atoms are saturated with hydrogen. Furthermore, the focus of these additional calculations is not the elucidation of the entire reaction pathway but only the important rate-determining steps. By comparing the ZnO/Cu interface with the ZnCu surface shown above, critical information on the catalytic activity of these structures can be concluded. We calculated the effect of the ZnO/Cu interface for the Cu(111) and Cu(100) surfaces on the binding energy of formate and the transition state (TS) toward H_2_COOH at the Zn-terminated edges of the ZnO stripes (see the SI for structures, Figures S10–S13). Given that the overall activity can be approximately determined by the free energy difference between formate and the transition state of H_2_COOH formation, we have focused on this difference for the ZnO/Cu interface and compared this to Cu(100), see Figure 7.

Interestingly, both formate and H_2_COOH bind at the interface of the ZnO/Cu(111) and ZnO/Cu(100) model in a similar fashion. Compared to formate on ZnCu(100) (see Figure 7), the energetic differences between the energy of the transition state toward H_2_COOH formation and the adsorption energy of formate are similar, with ZnO/Cu(111) and ZnO/Cu(100) being calculated to be slightly more active. Note also that ZnO/Cu(100) has a lower overall energy difference between HCOOH and TS-H_2_COOH compared to ZnO/Cu(111), again indicating that the (100) surface should have superior activity compared to (111). We stress though that the differences are rather small and within the accuracy of the employed DFT calculations.

### Infrared and Raman Spectroscopy

To gather further information on the structure of the pre-catalysts surface and to show the differences between the surfaces on the two catalysts (NCs vs NPs), the adsorption of CO was measured with DRIFTS at −187 °C. Prior to the experiments, the catalyst was reduced in 10% H_2_ in He at 250 °C to reproduce the pretreatment of the reactor experiments.

**Figure 8:**
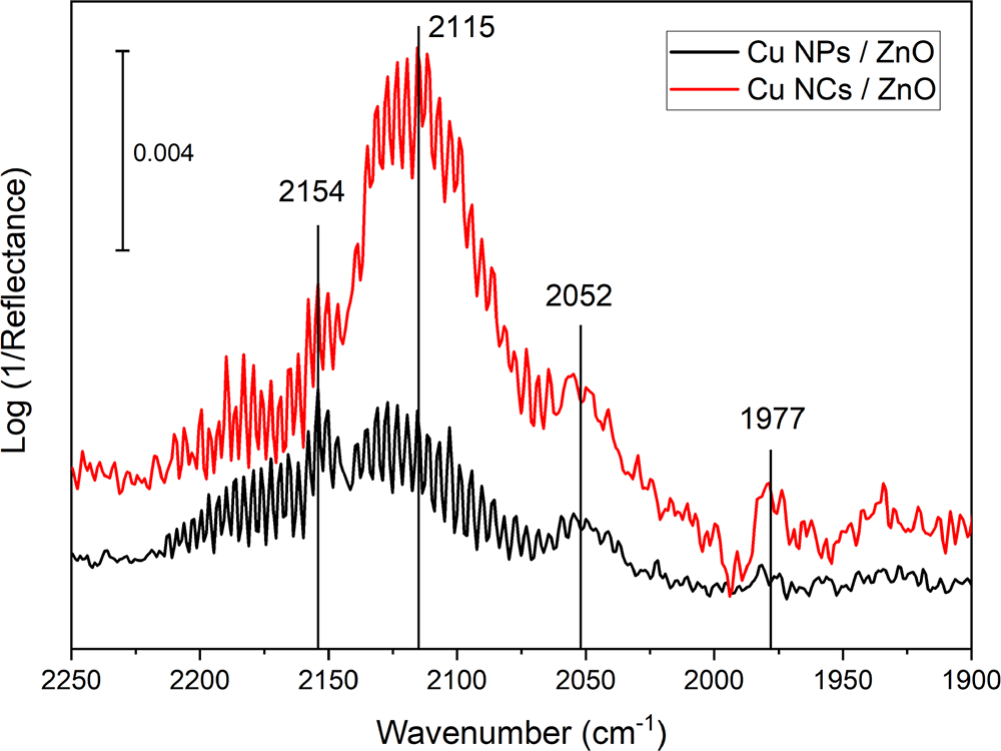
Comparison of the DRIFT spectra of Cu NCs and Cu NPs supported on ZnO in the presence of 1 mbar CO at −187 °C.

Figure 8 shows the spectra of adsorbed CO in the presence of an equilibrium pressure of about 1 mbar CO in the gas phase. Under these conditions, the bands of adsorbed species are superimposed by the vibrations of CO in the gas phase, which is evident from the rotational fine structure. A more detailed presentation of the spectra measured at multiple pressures during adsorption and desorption is provided in Figure S14.

Bands in the range from 2220 to 2150 cm^–1^ are typically assigned to Cu^2+^–CO, while Cu^+^–CO bands are in the range of 2160–2080 cm^–1^ and those found <2130 cm^–1^ are assigned to Cu^0^–CO.^63^ Bands due to CO adsorption on Zn^2+^ sites (normally located at 2180–2190 cm^–1^) are not clearly observed. The bands around 2150 cm^–1^ can be explained by the CO interaction with surface OH groups.^63^ The small band detected at 2052 cm^–1^ is due to CO linearly adsorbed on terrace sites of low-indexed Cu surface planes. The most striking difference between the two pre-catalysts is the band at 2115 cm^–1^, which is much more pronounced in the spectrum of CO adsorption on Cu-NCs. The peak can be attributed to Zn^δ+^–CO, Cu^δ+^–CO, or CO adsorbed on highly defective Cu(0).^64^ A band at 1977 cm^–1^, which appears more clearly in the spectrum of the Cu NCs, could come from the Cu–ZnO interface,^65^ originating from a CO molecule adsorbed in a bridged mode between metallic Cu and a Zn site on ZnO. The presence of the band reveals a strong interaction between Cu an ZnO. Since, as we will show below, the presence of ZnO on the Cu surface has been revealed by Raman spectroscopy, the IR band at 2115 cm^–1^ is mainly assigned to the CO–Zn^δ+^, without ruling out the complete absence of cationic Cu species or dissolved oxygen in our oxide-derived catalysts, especially in the case of the Cu_2_O nanocubes. The band position is also in agreement with the calculated stretching frequency of CO adsorbed on graphite-like ZnO bilayers.^66^

As a result from these experiments, we can conclude that the two pre-catalysts have a different surface structure. Furthermore, the features assigned to the Cu–ZnO interface suggest that this might be a significant part of the catalyst surface and probably even more essential than the different Cu surfaces. Indeed, almost all features visible in the IR spectra can be assigned to some sort of ZnO or an unusual copper oxide site. The idea of oxidized copper is not new and was already proposed in older literature.^67,68^ This may be especially important if oxygen plays a critical role in the active site for this reaction at the interface of Cu and Zn (Cu–O–Zn).^53^ The migration of Zn from the support on the Cu particles and the formation of a ZnO overlayer was also reported before^58^ and already suggested during the discussion of the reactivity data. Furthermore, the presence of minority copper oxide sites (or dissolved oxygen in Cu) is a possibility that should not be excluded. Nonetheless, the initial shape of the Cu pre-catalyst supported on ZnO might affect the ZnO encapsulation process taking place during the activation state and under reaction conditions due to the initially different Cu/ZnO contact areas and surface structures. As the activity of Cu for the hydrogenation of CO_2_ to methanol is heavily dependent on the presence of Zn, these surface sites at the Cu–ZnO interface are probably the most important ones.

**Figure 9:**
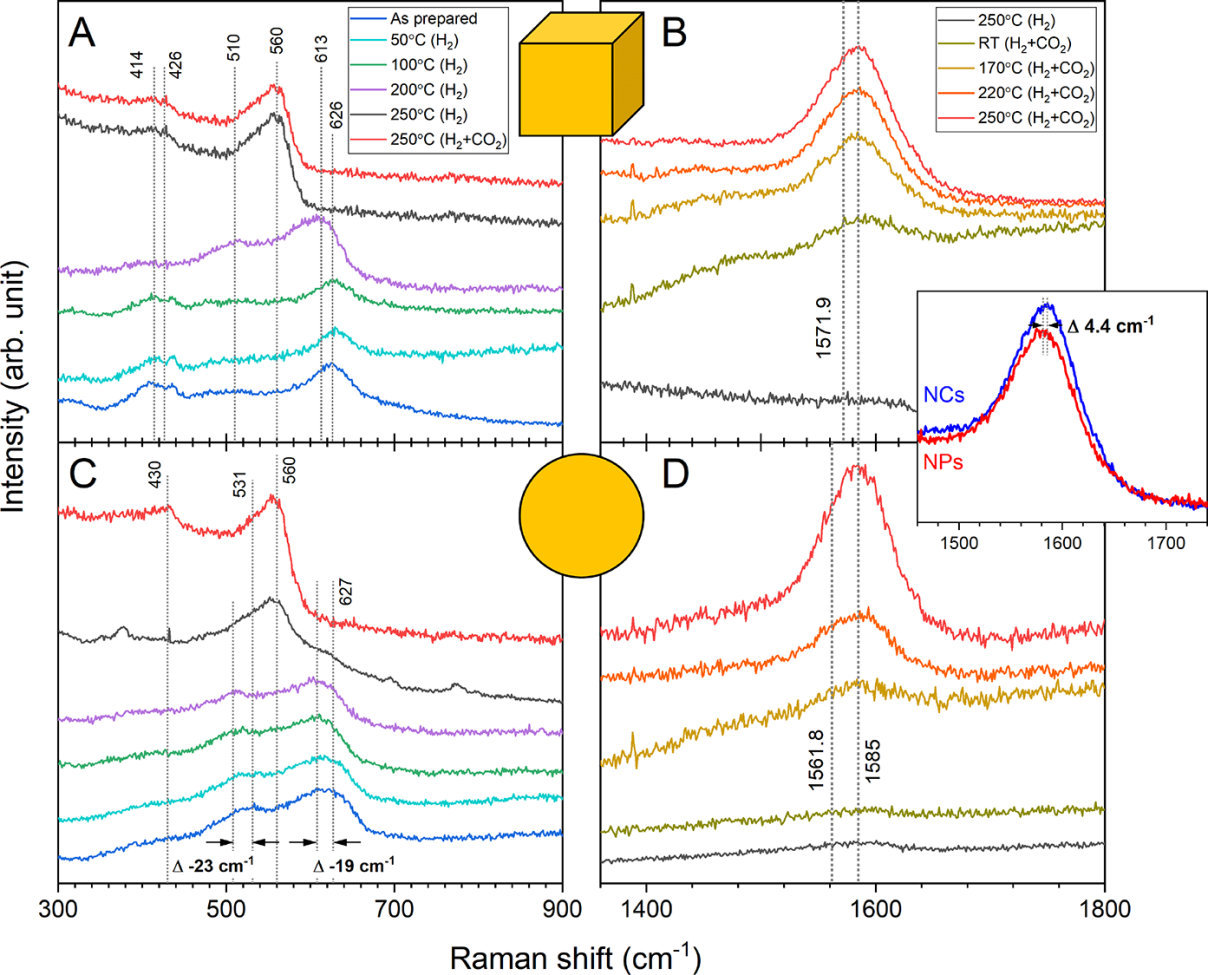
*In situ* Raman spectroscopy on the Cu NCs (A, B) and spherical NPs (C, D), both supported on ZnO. (A, C) Spectra acquired during the reduction in H_2_ with increasing temperature and under reaction conditions at 250 °C. The lines correspond to Cu^2+^ (626 cm^–1^), Cu^+^ (510 cm^–1^, 613 cm^–1^), and ZnO (426 cm^–1^, 560 cm^–1^). (B, D) Raman spectra of the formate region at the end of the reduction (10% H_2_ in He) and under reaction conditions (60% H_2_ + 20% CO_2_ + 20% He, 15 bar) at multiple temperatures showing the increasing formate band with temperature. The inset shows the spectra for the cubic and spherical pre-catalysts compared directly under reaction conditions. The peak position of the NP catalyst is shifted to slightly lower wavenumbers.

To further investigate the surface structure and composition under actual reaction conditions, *in situ* Raman spectroscopy was performed. The Cu NCs and the NPs were first measured in their initial state. The cubes (Figure 9A) show peaks corresponding to Cu^2+^ (626 cm^–1^)^69^ and ZnO (426 cm^–1^, 576 cm^–1^).^70^ This in line with the previous XPS characterization (Figure S2) that showed a CuO layer forming on top of the Cu_2_O cubes due to the exposure to air after the synthesis. The spherical NPs (Figure 9C) show additionally features from Cu^+^ (510 cm^–1^, 613 cm^–1^),^71^ indicating that this catalyst is slightly more reduced on the surface from the start. The samples show a typical downshift of the vibrational frequencies in comparison to bulk references caused by the nanosized character of these materials.^69^

When introducing H_2_ for the reduction of the catalyst and during annealing, gradual changes are observed for both samples. Upon reaching 200 °C, the Cu^2+^ becomes reduced to Cu^+^ on the NCs. Once at 250 °C, only the fingerprint of ZnO can be observed, indicating the complete reduction of Cu so that only metallic copper remains on the surface. For the spherical NPs, a downshift of the peaks is first observed, indicating the transition from a mixture of Cu^2+^ and Cu^+^ to only Cu^+^. Subsequently, the contributions from the copper oxides disappear from the surface, indicating the complete reduction to metallic Cu, with only the ZnO fingerprint remaining. The ZnO feature at 560 cm^–1^ is unusually strong in both samples and suggests the existence of oxygen vacancies in the ZnO structure.^70^

When switching to the CO_2_ + H_2_ mixture, no further changes are observed in the vibrations corresponding to Cu or ZnO. Nonetheless, a band between 1500 and 1660 cm^–1^ corresponding to formate^72^ can be observed for the NCs as well as for the NPs (Figure 9B). The band is not present without CO_2_ and increases with increasing reaction temperature. This is consistent with other literature reports discussing formate as the most important intermediate for this reaction.^73^ A direct comparison of the formate region for the cubic and spherical particles is shown in Figure 9 (inset) for the normalized spectra. There is a slight difference in the peak position of the formate band for the NCs and NPs under reaction conditions. Note that the formate band that is observed here is quite broad and therefore probably corresponds to a collection of multiple components originating from formate being present adsorbed on multiple different surface sites. The prevalence of the individual components should still make a difference in the band shape and position, which allows us to distinguish between the different catalysts and make a statement about the surface structure. For comparison, theoretical values for the asymmetric vibrational mode of formate on different surfaces were obtained by using DFT calculations (Table 1). This difference observed experimentally between NCs and NPs is consistent with the assumption that a bigger fraction of the surface on the NCs has a (100) termination as compared to the spherical NPs that would be dominated by (111) surface planes. Additionally, the overall position of the peaks indicates that formate is likely to be adsorbed not on pristine metallic Cu surfaces but on a ZnO-covered Cu surface for both samples. The actually measured peak positions are even slightly higher than the calculated ones for the ZnO covered Cu surfaces. Here, it should be noted that in the theoretical calculations, a graphitic layer of ZnO was assumed, but the real state will probably be more complex. Nevertheless, we take this model as an approximation and together with the experimental results, this confirms the presence of a ZnO covered surface under reaction conditions since the simple Cu or CuZn alloy surfaces should have been observed at lower vibrational frequencies. The theoretical difference between the vibrational frequencies of formate adsorbed on ZnO-covered Cu(111) and Cu(100) is about 10 cm^–1^. The shift in the experimental data is smaller (4.4 cm^–1^), which is most likely due to the restructuring of the surfaces of the NCs under reaction conditions (cube rounding) that was observed by TEM. As stated above, we consider that the catalysts have a mixture of multiple facets, but still, because of its initial shape, the presence of Cu(100) facets is still more prevalent on the NC catalyst. Therefore, the difference in the band position is still visible, but decreased.

**Table 1:** Theoretical Values for the Raman Shift Frequencies Corresponding to the Formate Intermediate Adsorbed on Different Cu, Cu_2_O, CuZn, and ZnO/Cu Surfaces

surface.	vibr. freq. (cm^–1^)
Cu(111)	1519
Cu(100)	1525
CuZn(100)	1527
Cu(211)	1530
CuZn(211)	1537
ZnO/Cu(111)	1562
ZnO/Cu(100)	1572
ZnO(100)	1550
Cu_2_O(100)	1544
Cu_2_O(110)	1544

Finally, our synergistic experimental and theoretical study highlights the importance of the initial pre-catalyst structure (particle size, shape, composition, and contact area with the support) on the subsequent evolution of the catalyst under reaction conditions and the resulting activity and selectivity trends.

## Conclusions

Cubic Cu_2_O and spherical Cu particles supported on ZnO were used as model systems to investigate the role of the pre-catalyst structure on the CO_2_ hydrogenation reaction for the synthesis of methanol. Despite the fact that both pre-catalysts experienced drastic structural and chemical changes in the course of the reaction, including the rounding and reduction of the Cu_2_O nanocubes, the reduction of the CuO_*x*_ spherical NPs, their sintering, and the encapsulation of both by ZnO, distinct activity and selectivity trends were obtained. In particular, enhanced methanol yields were found for the cubic pre-catalysts while keeping a similar selectivity to the spherical NPs.

More specifically, it was observed that the initial reduction of the copper in hydrogen affects the cubic shape, but that the main morphological changes take place under CO_2_ + H_2_ reactions conditions, with significant NP rounding visible as a function of the time on stream and sintering at temperatures >250 °C, which was associated with a decrease in the activity. Nonetheless, it is plausible that smaller {100} domains still remain and that such orientation is preferred when the pre-catalyst was initially cubic as compared to reference spherical NPs of similar size. Importantly, we propose that the initial surface of the Cu particle and contact area with the support is critical for the formation of the Cu–ZnO interface, which will lead to the creation of the active site. Furthermore, our DFT calculations point to the rather favorable CO_2_ hydrogenation to methanol on the ZnO-covered Cu(100) surface. One big remaining challenge here is to identify the exact nature of Zn in the active site. It seems that the trend observed here holds true for both ZnCu and ZnO/Cu, with the experiments however suggesting ZnO overcoating being more likely. Alternatively, also states in between, including defective ZnO_*x*_, may be present under reaction conditions. Nevertheless, our results clearly point to the superior catalytic performance for methanol synthesis of the (100) surface compared to (111).

To better put our work into perspective as compared to the traditional Cu/ZnO systems made by co-precipitation for practical applications, the following observations are made. Successful catalysts^33,50^ expose the (111) facet toward its mineral spacer ZnO and the (100) facet to the overgrown ZnO. Careful activation of the hydroxo-carbonate precursor leads to highly active materials with Cu particles exposing (100) facets, plus rounded edges^74^ as the predominant morphology. The similarity in these observations is striking even when the precipitated catalysts exhibit a particle size lower by an order of magnitude. The present work highlights a function of the ZnO component not so much discussed so far: encapsulation or stabilization of the surface of Cu nanostructures by ZnO helps to preserve the Cu morphology even under the highly reducing synthesis conditions. Brass formation may be avoided by traces of oxygen stemming from the reduction of CO_2_. The detrimental effect of brass formation upon overheating the binary system (Cu/ZnO) documented in Figure 5 and echoed in the DFT calculations is pointing in this direction as well. This is fully analogous to observations made with precipitated catalysts^53^ that lose their high productivity upon brass formation. Whether the brass electronic structure or the loss of the Cu(100) surface orientation is the more negative effect remains hard to decide based upon our theoretical results.

The experimental results presented in this work pointing toward the non-perfect nature of the copper metal phase in the active catalysts (Figures 3, 8, and 9) are of relevance to explain the driving force of the kinetic transformations of the Cu component during activation and reaction. It is the tendency of the system to remove structural imperfections within the lattice (stacking faults) and at the subsurface (residual oxygen) that provide the energy for the microstructural changes observed. Also, this behavior of “methanol copper” is fully in line with the observations on technical catalysts^75^ used for CO_2_ hydrogenation. The present work provides solid and complementary evidence for the non-perfect nature of active Cu. It indicates that it may not only be the structure sensitivity of the adsorbate-Cu metal interaction, but the gain or loss of the imperfection of the Cu that makes the difference between highly active and less active variants of the same system. This might occur while creating the active sites for the synergistic interaction between Cu and ZnO without brass formation.

For quality control and stability improvement of technical catalysts, the present study shows that after the activation, the abundance of particles being stepped and/or exposing (100) facets indicates high performance, whereas the abundance of smooth rounded particles stands for a less active systems. This is a valuable descriptor as it can be quantified with reasonable effort. The use of the intensity ratio of features of adsorbed CO as shown in Figure 8 may be an even more attractive proxy for counting the active surface of an activated or used catalyst. The simplistic idea that deactivation of technical catalysts is due to “sintering”, understood as a combination of Cu metal nanostructures and concurrent loss of the metal surface area is oversimplified as illustrated in the present work.

The study illustrates a strategic experimental approach for the identification of the function of specific surface structures. The obtained knowledge can be used for the improvement and design of highly efficient catalysts with pre-defined morphological features. To this end, the unit operations of the technical synthesis, namely, oxidation of the carbonate to oxide and its reductive activation, can be optimized by using the spectroscopic descriptors of this work. During oxidation, care has to be taken to optimize the phase separation into Cu as oxide and Zn carbonate, such as to maximize their contact area. The atmosphere and temperature program of the pre-treatment and catalytic process are variables amenable to descriptor-controlled modifications. The reduction step can be designed according to the present results such as to retain a small amount of oxygen in the bulk metal matrix and to minimize the temperature load during formation of the Cu phase in order to at least preserve some of the favorable faceting of the shaped pre-catalyst structure, in addition to its contact to the Zn oxide phase.

With respect to the composition of the catalyst formulation, in this case Cu-ZnO_1–*x*_, it may be advisable to add the function of an oxygen storage phase to the X-component modifying the defect structure and conductivity of the ZnO. The addition of an element like Zr or Ce in amounts preventing the formation of acid–base active species (giving rise to DME formation) could be an option. The latter could support the retention of the structural modifier oxygen within a bulk metal phase under high partial pressures of hydrogen moderated in their reductive chemical potential by the reaction product water. The addition of a Zn-oxide component may also harden the system against hydrolytic attack during use in pure CO_2_ reduction. Here, the formation of Zn carbonate or hydroxycarbonate would retract the intimate interaction of the contact Cu–ZnO_1–*x*_ and hence destroy the synergy of the two components.

Finally, this work presents reference spectroscopic data and analytical descriptors enabling the rational experimental design when optimizing the amount and spatial distribution of residual oxygen and stabilizing the additives in novel catalysts. One should aim to maximize and stabilize rough metal terminations with (100) orientation of Cu nanoparticles in overgrowth of ZnO_1–*x*_.

## Supporting Information Available

Tables with ICP-MS and N_2_O-RFC results, additional STEM images with size histogram and EDX spectra, XPS spectra, Cu oxidation state and crystallite size extracted from XRD of Cu NPs on ZnO, additional XAS and DRIFTS spectra, additional reactivity results (MeOH yield normalized by Cu content, time dependent MeOH production, H_2_ consumption during reduction), additional DFT (methods, models, and Gibbs free energy of the intermediates and transition state) (PDF)
