# Supplementary material for: Shape-Dependent CO2 Hydrogenation to Methanol over Cu2O Nanocubes Supported on ZnO
Source: J Am Chem Soc. 2023 Jan 30;145(5):3016–30. doi: 10.1021/jacs.2c11540 (PMC9912329; doi:10.1021/jacs.2c11540)
Supplement: Supplementary file 1 — ja2c11540_si_001.pdf [file ja2c11540_si_001.pdf]

Supporting information for

Shape-Dependent CO_2 Hydrogenation to Methanol over Cu_2O Nanocubes supported on ZnO

David Kordus[‡,†], Jelena Jelic[§], Mauricio Lopez Luna[‡], Núria J. Divins[†,⊥], Janis Timoshenko[‡], See Wee Chee[‡], Clara Rettenmaier[‡], Jutta Kröhnert[$], Stefanie Kühl[‡], Annette Trunschke[$], Robert Schlögl[$], Felix Studt[§,‖,] and Beatriz Roldan Cuenya[‡]**

[‡] Department of Interface Science, Fritz-Haber Institute of the Max Planck Society, Berlin 14195, Germany

[†] Department of Physics, Ruhr University Bochum, 44780 Bochum, Germany

[§] Institute of Catalysis Research and Technology, Karlsruher Institute of Technology, 76344 Eggenstein-Leopoldshafen, Germany

[$] Department of Inorganic Chemistry, Fritz-Haber Institute of the Max Planck Society, 14195 Berlin, Germany

[‖] Institute for Chemical Technology and Polymer Chemistry, Karlsruhe Institute of Technology, 76131 Karlsruhe, Germany

[⊥] Current address: Institute of Energy Technologies, Universitat Politècnica de Catalunya, 08019 Barcelona, Spain

* Corresponding authors: roldan@fhi-berlin.mpg.de; felix.studt@kit.edu

ICP-MS

**Catalyst**	**Cu/(Cu+Zn)**
Cu_2O cubes / ZnO<br>As prepared	31.0 %
Cu_2O cubes / ZnO<br>After reaction	30.7 %
Cu NPs / ZnO<br>As prepared	34.2 %
Cu NPs / ZnO<br>After reaction	34.3 %
Commercial reference<br>(Alfa Aesar)<br>As prepared	73.6 %

Table S1 Atomic fraction of Cu and Zn in each catalyst as determined by ICP-MS analysis before and after reaction (60% H_2 + 20% CO_2 + 20% He, p = 60 bar, T = 250°C). Values only take into account the contribution of Cu and Zn. The commercial reference included Mg and Al as well.

N_2O-RFC

**Catalyst**	**Cu surface Area ($m^2/g_{catalyst}$)**
Cu_2O cubes / ZnO	1.60
Cu NPs / ZnO	1.57
Commercial reference (Alfa Aesar)	13.31

Table S2 Specific Cu surface area as determined by N_2O reactive frontal chromatography.

STEM/EDX

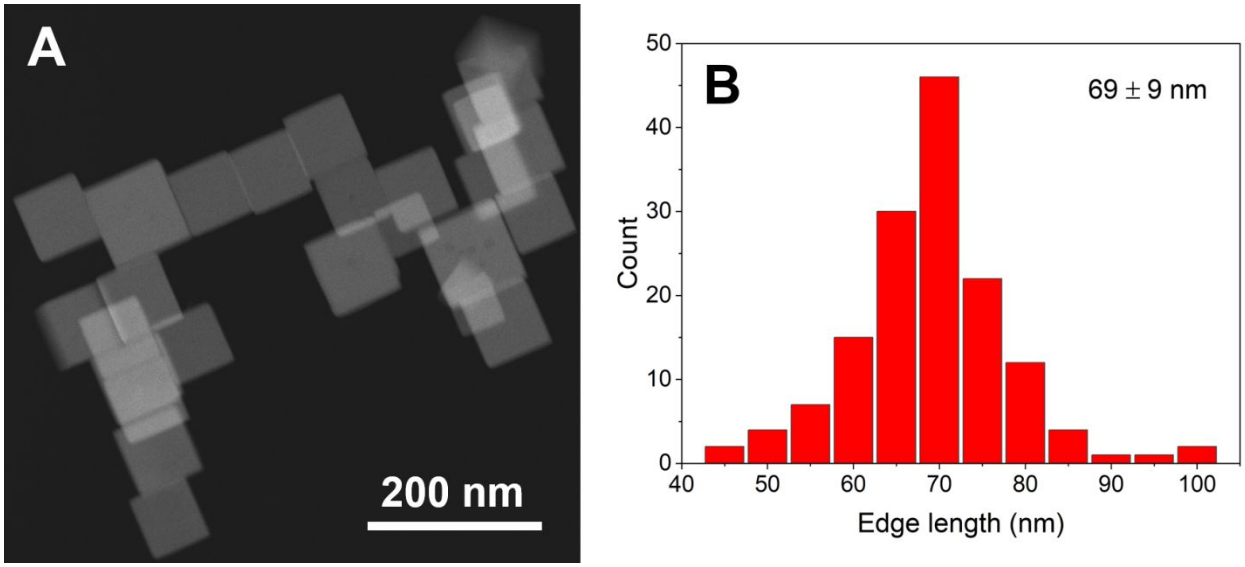

Fig. S1 (A) Exemplary STEM image of the bare Cu_2O nanocubes. (B) Histogram for the cube size extracted from multiple images.

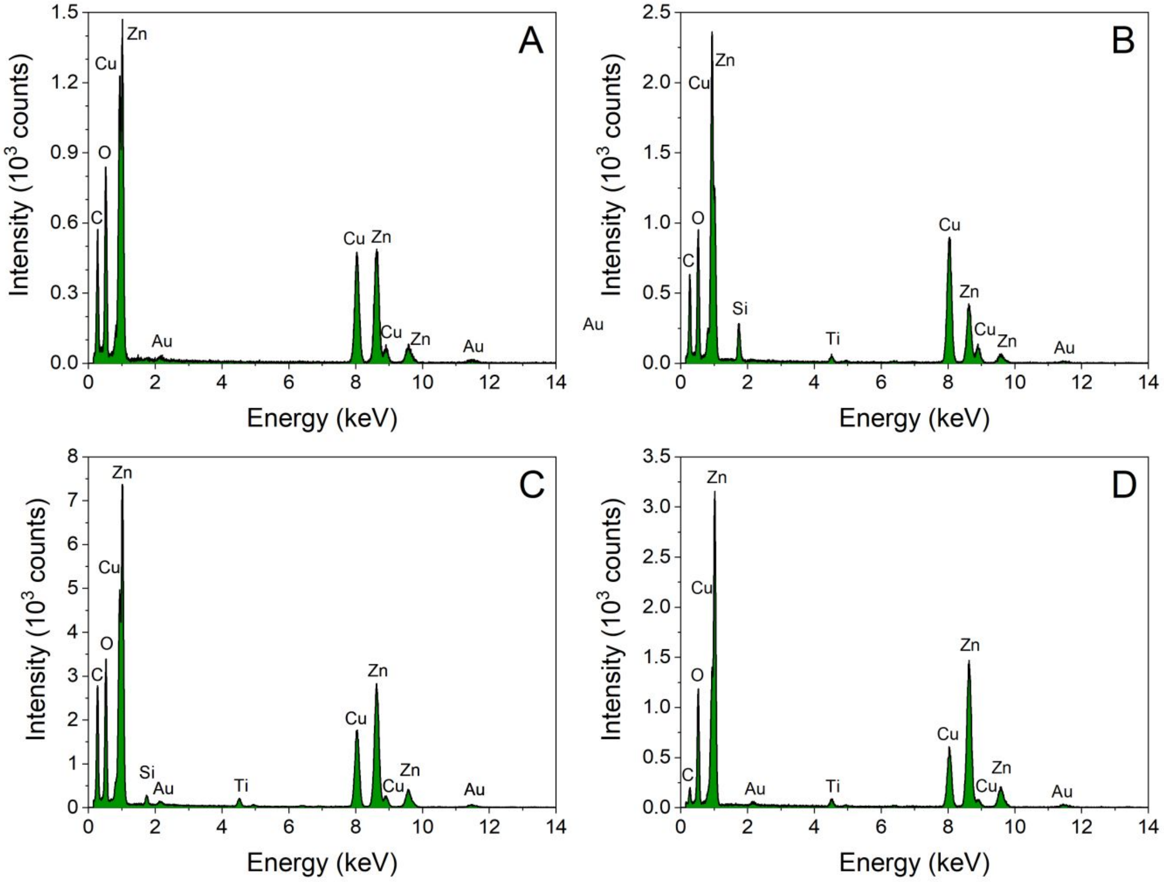

Fig. S2 EDX spectra corresponding to the EDX maps shown in figure 1 in the main text. (A) As prepared catalyst, (B) after reduction in H_2 at 170 ℃ for 2 h and after the CO_2 hydrogenation reaction at 170 ℃ for (C) 10 min and (D) over 100 h. The main contributions originate from Cu, Zn and O. Additional contributions (Au, C) originate from the background of the sample holder/grid/chamber. Some of the spectra after reaction/reduction show also a Si peak, because of some quartz wool pieces that were used to fixate the catalytic bed.

XPS

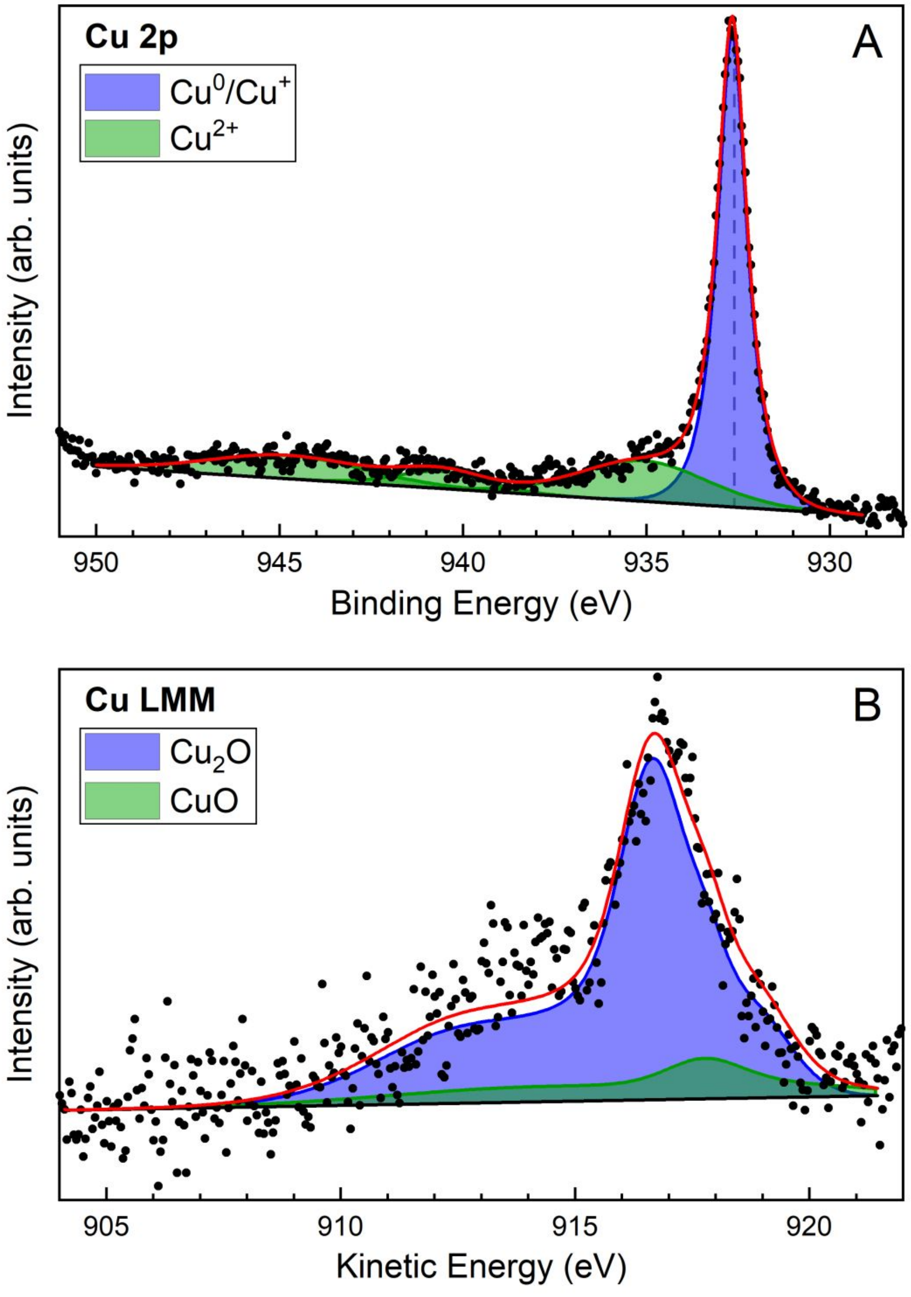

Fig. S3 XPS spectra of the of the (A) Cu $2p_{3/2}$ and (B) Cu LMM regions of the as prepared copper oxide NCs. The Cu $2p_{3/2}$ shows Cu^0/Cu^+ as the main contribution. The Cu LMM Auger region reveals that Cu_2O species dominate the spectrum. Fitting of the Cu Auger region resulted in 85% Cu_2O and 15% CuO.

XRD

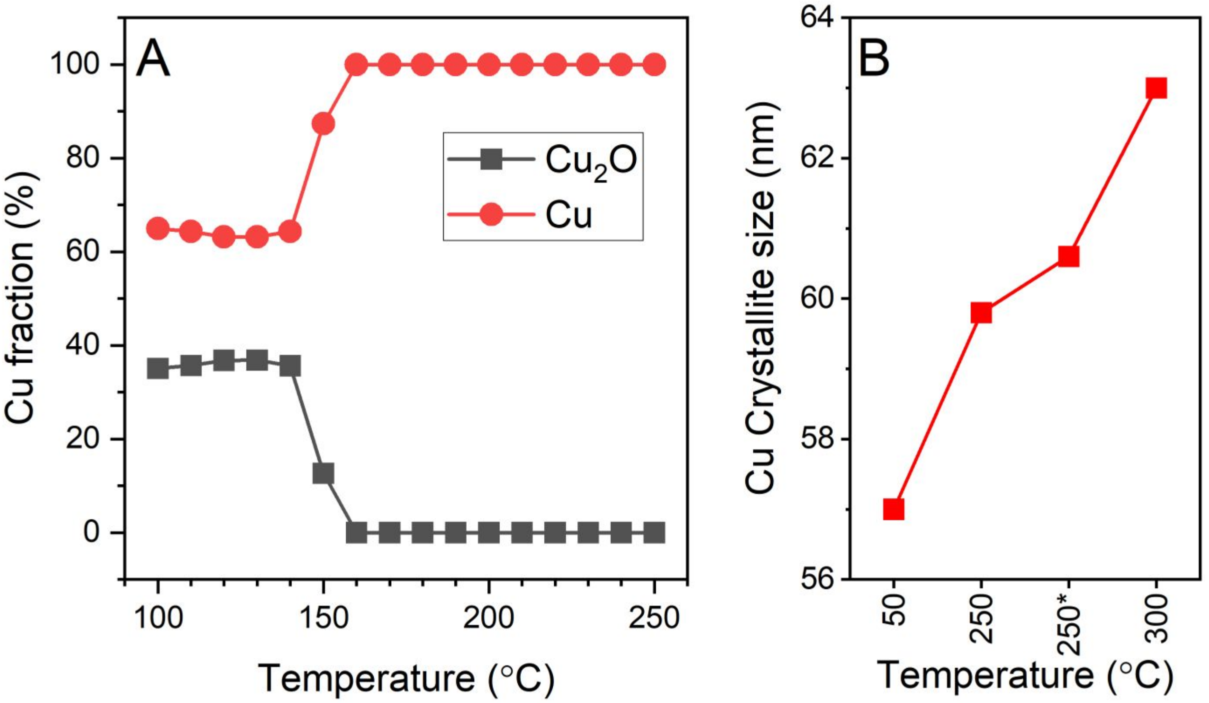

Fig. S4 Results from i*n situ* XRD measurements of commercial Cu NPs supported on ZnO. (A) Evolution of the oxidation state of Cu during the reduction treatment. (B) Cu crystallite size extracted by Rietveld fitting analysis for the catalysts under reaction conditions (75% H_2 + 25% CO_2, p = 10 bar) at the indicated temperatures. The point labeled 250* represents a second scan at 250°C and therefore shows the changes over time.

XAS

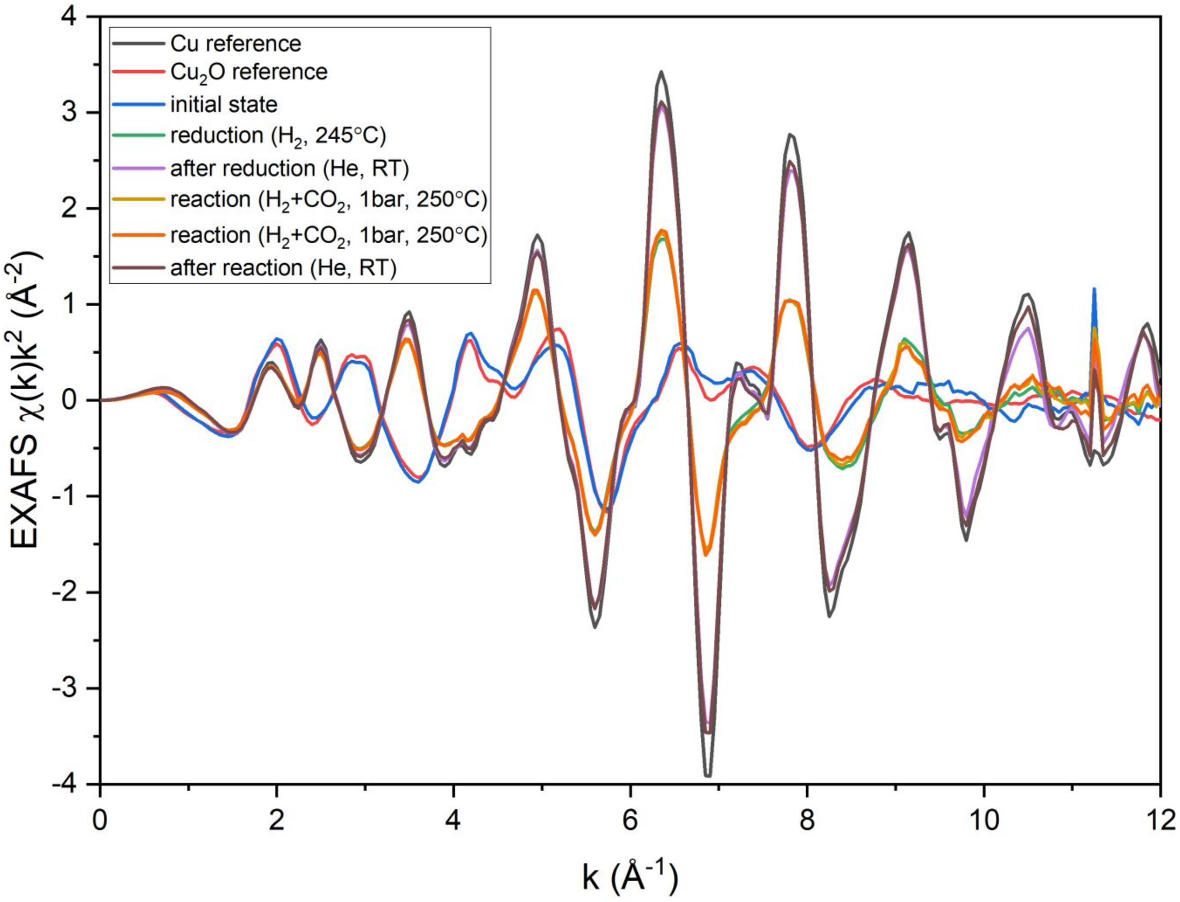

Fig. S5 *Operando* EXAFS spectra of the Cu-K edge of the Cu_2O NCs/ZnO catalyst during and in between the different treatments as indicated on the figure. Additionally reference spectra of Cu_2O and Cu are displayed.

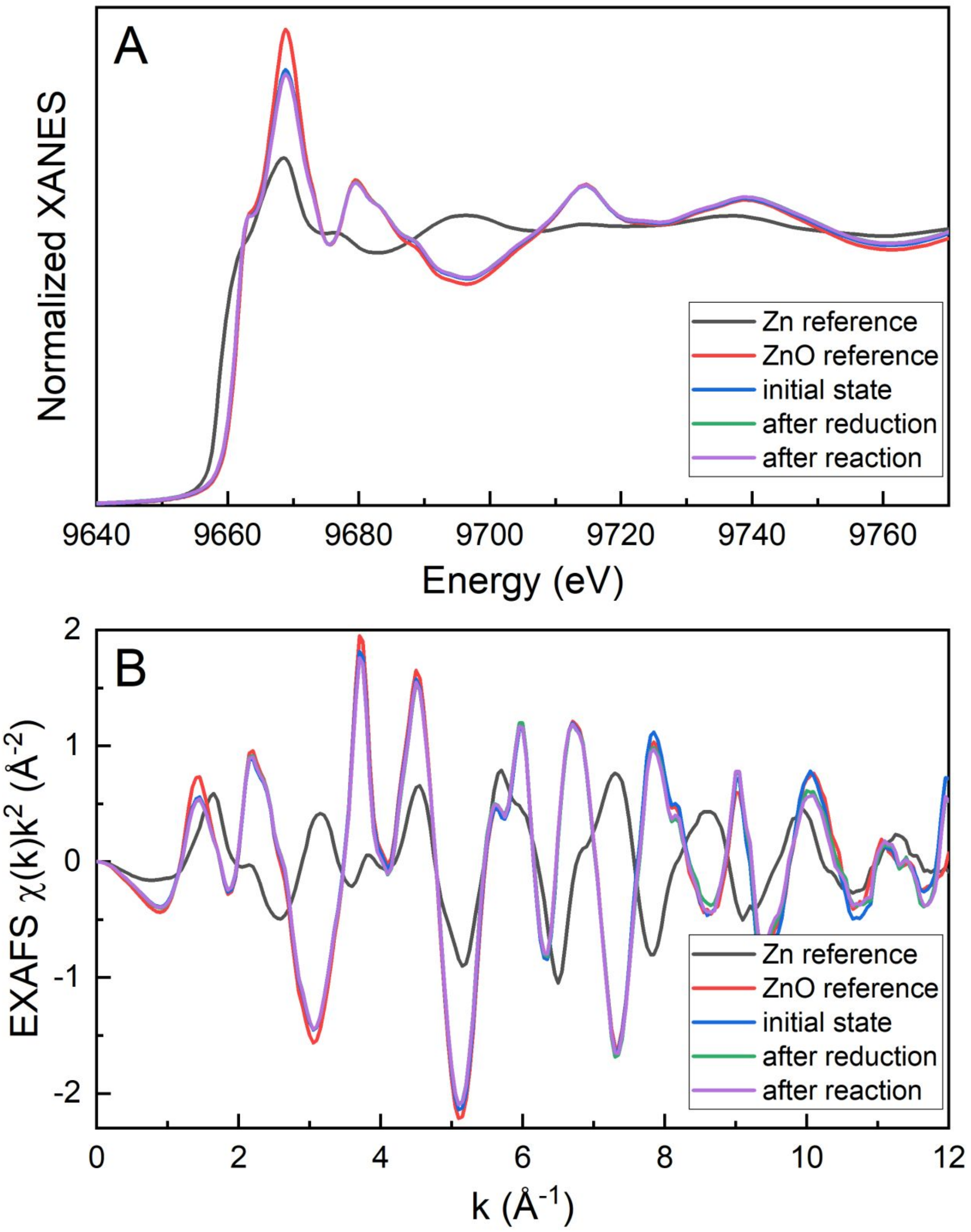

Fig. S6 *Operando* (A) XANES and (B) EXAFS spectra of the Zn-K edge of the Cu_2O NCs/ZnO catalyst in the initial state and after reduction (20% H_2 in He, 245°C) and reaction (75% H_2 + 25% CO_2, 250°C) treatments. Additionally reference spectra of ZnO and Zn are displayed.

REACTIVTY

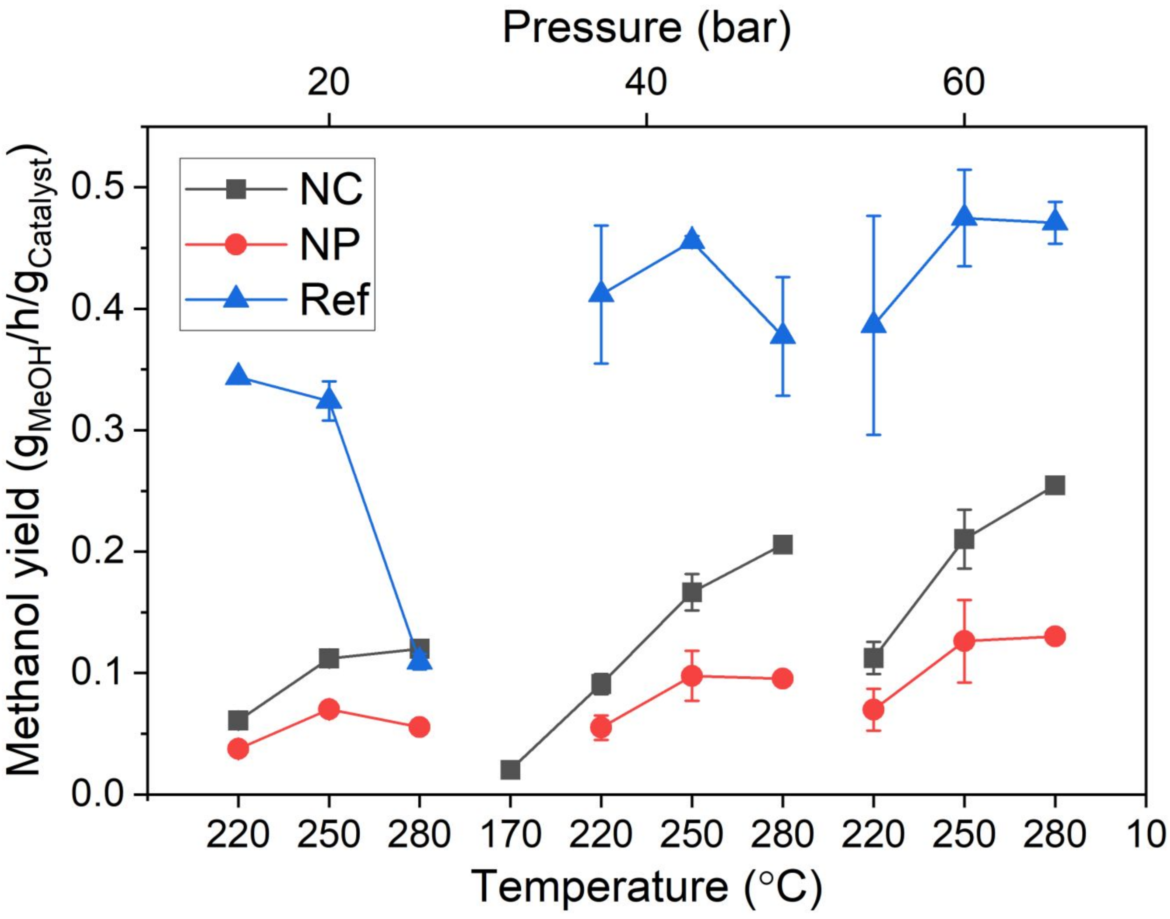

Fig. S7 Methanol yield normalized per gram of catalyst for the Cu_2O nanocubes (NCs) on ZnO, spherical Cu NPs on ZnO (NP) and the commercial reference catalyst (CR).

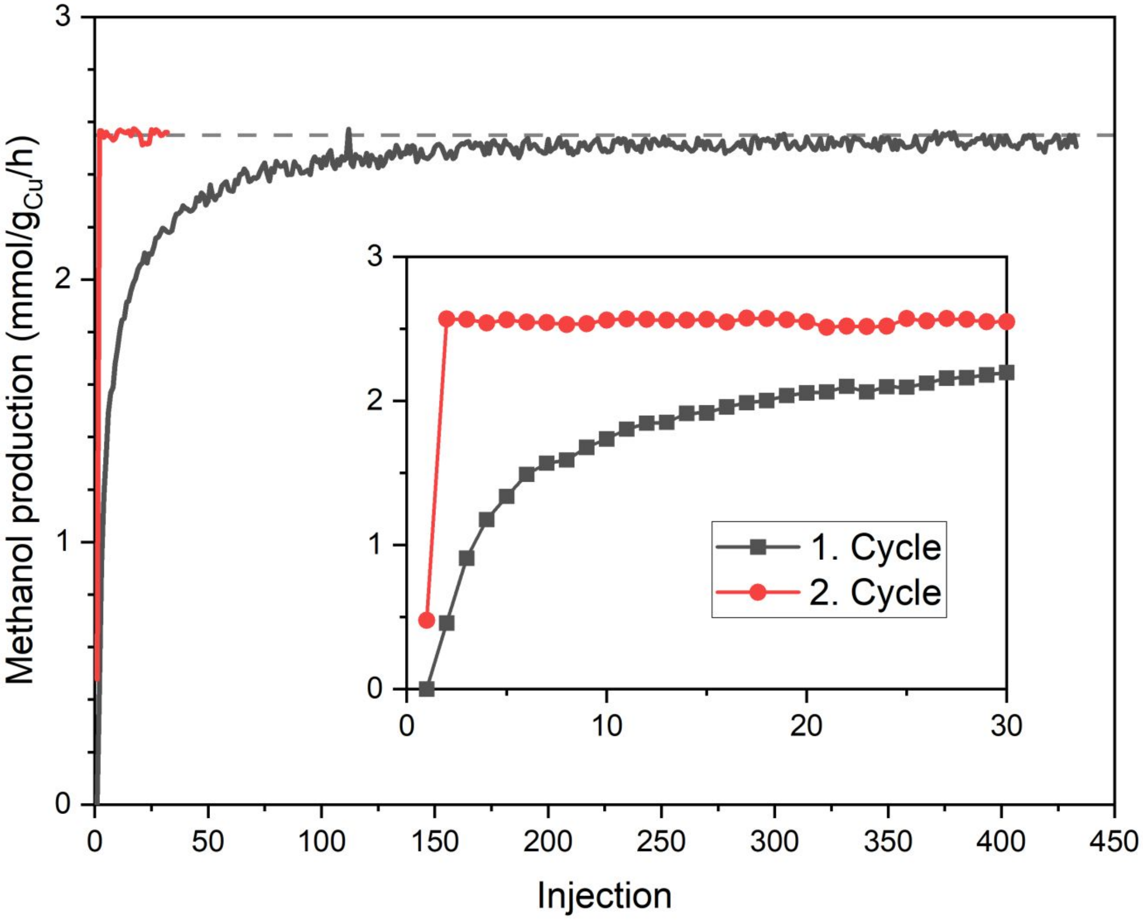

Fig. S8 Methanol production rate during the start of the reaction at p = 40 bar and T = 170°C. During the first cycle the reaction rate slowly increases. The second cycle was performed on the same catalyst after the first cycle and flushing the reactor with He in between cycles. One injection happens around every 20 minutes.

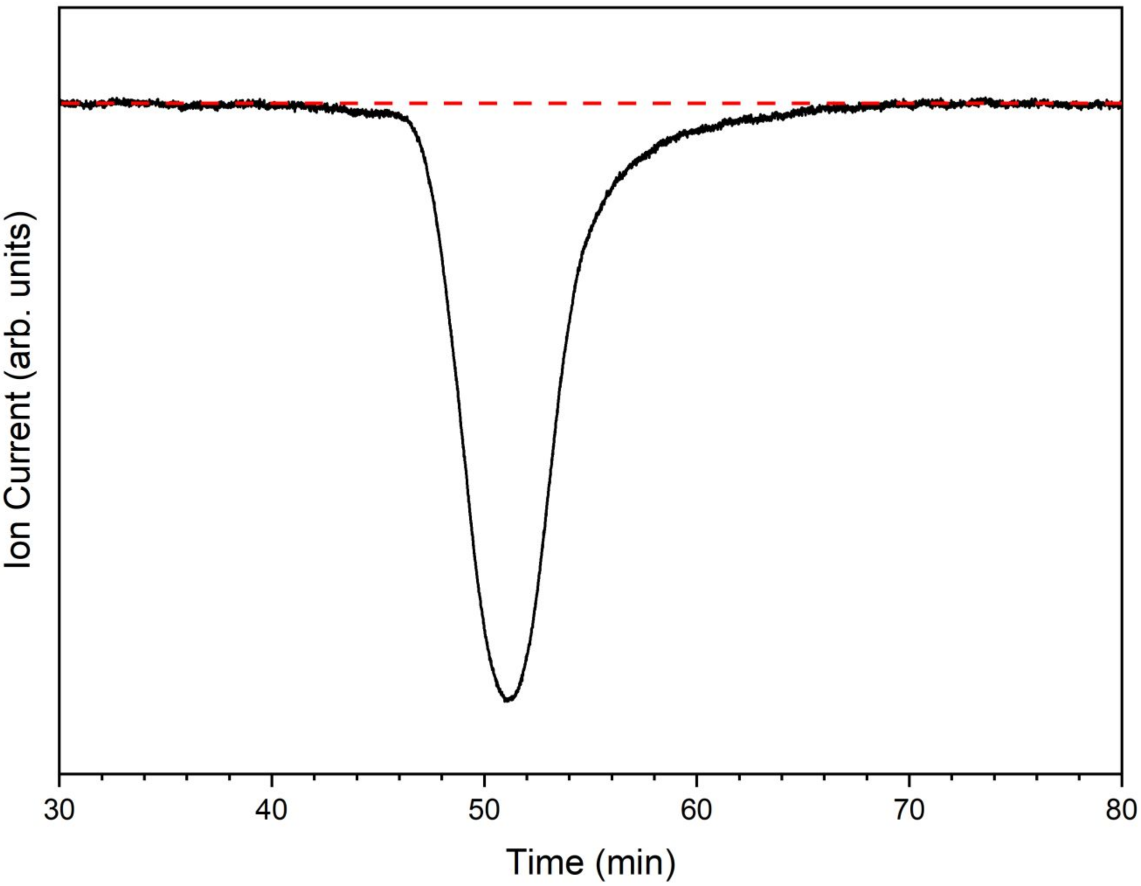

Fig. S9 H_2 concentration as seen by mass spectrometry during the reduction of the catalyst. The negative peak represents the H_2 consumption because of the reduction of Cu_2O to metallic Copper. The red dashed line is a visual aid.

DFT

ZnO single layer without support is stabilized in flat, so-called graphitic form.

Full ZnO layer over Cu(111) is modeled using 3x3-ZnO unit placed over 4x4-Cu(111)[1] and the adhesion energy between ZnO in the gas phase and the Cu surface is -0.16 eV per ZnO unit. Truncated ZnO/Cu(111) system has been modeled using four layers thick 7x4 large Cu(111) unit cell with 3x3 large single layer ZnO (Figure S10). Adhesion energy for this system -0.02 eV. Two types of interfaces are present, oxygen-terminated (called A) and Zn-terminated (called B) interface. Hydrogenation of oxygen atoms at the A interface is favorable for the given gas phase conditions. Due to OH groups present between the Zn and Cu surface at the interface A, interface B (ZnO^B/Cu(111)) has been chosen for the further studies.

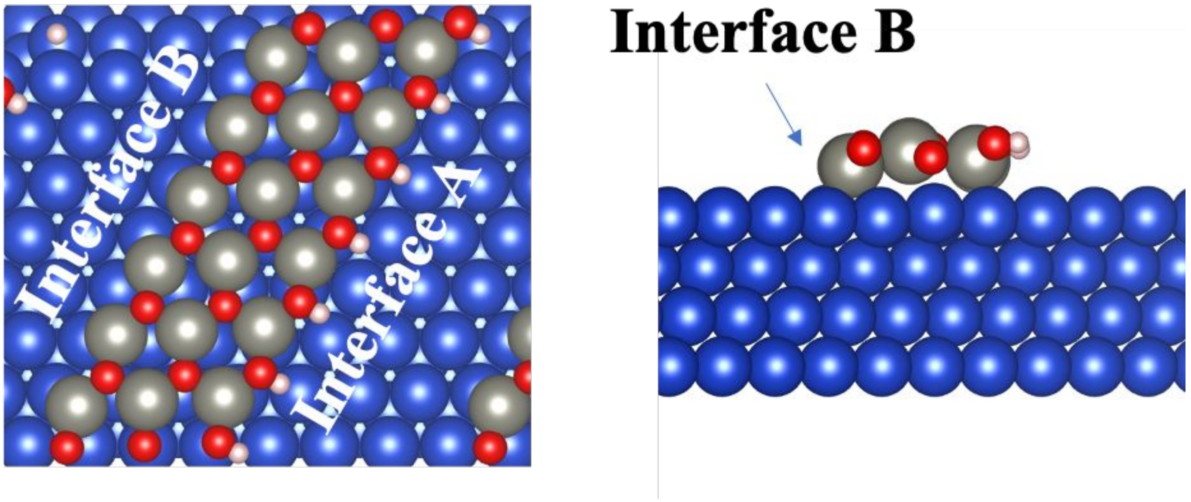

Fig. S10 3x3-ZnO/7x4-Cu(111) model system (top and side view)

The full single layer ZnO over Cu(100) was modeled using 3x2-ZnO layer over 4x2 large Cu(100) and adhesion energy is -0.20 eV. The truncated ZnO/Cu(100) system has been modeled using four layer thick 4x6 large Cu(100) unit cell with 3x3 large single layer ZnO nanowire (truncated in *y* direction and infinite in *x* direction). Same type of interfaces A (additionally hydrogenated) and B

[1] *ChemCatChem* **2021**, *13*, 4120 – 4132

are present (Figure S11). Adhesion energy for this system is -0.07 eV, slightly larger as compared to the ZnO over Cu(111).

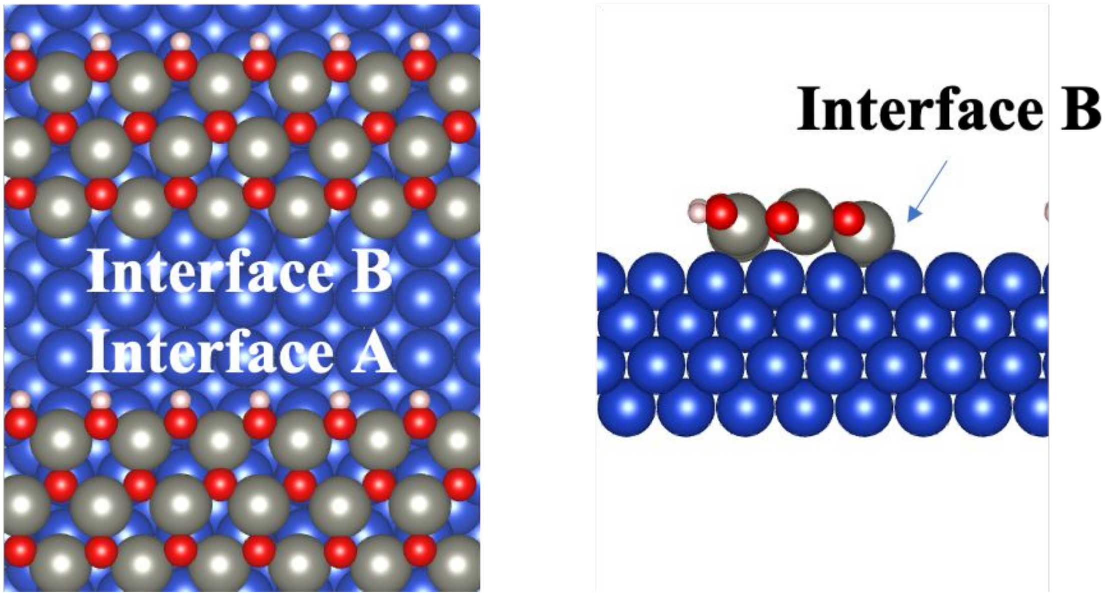

Fig. S11 3x3-ZnO/4x6-Cu(100) model system (top and side view)

Gibbs free energy of the CO_2 hydrogenation intermediates and H_2COOH transition state calculated for the interfaces B are given in Table S3:

ΔG (eV)	ZnO^B/Cu(111)	ZnO^B/Cu(100)
HCOO	-0.11	-0.17
H_2COOH	0.98	0.78
TS-H_2COOH	1.50	1.35
OH	0.34	0.13
OCH_3	0.29	0.10

Table S3 Gibbs free energy of the CO_2 hydrogenation intermediates and H_2COOH transition state calculated for the interfaces B at $T = 500$ K, $p(CO_2) = 18$ bar, $p(H_2) = 3$ bar, $p(H_2O) = p(CH_3OH) = 1$ bar

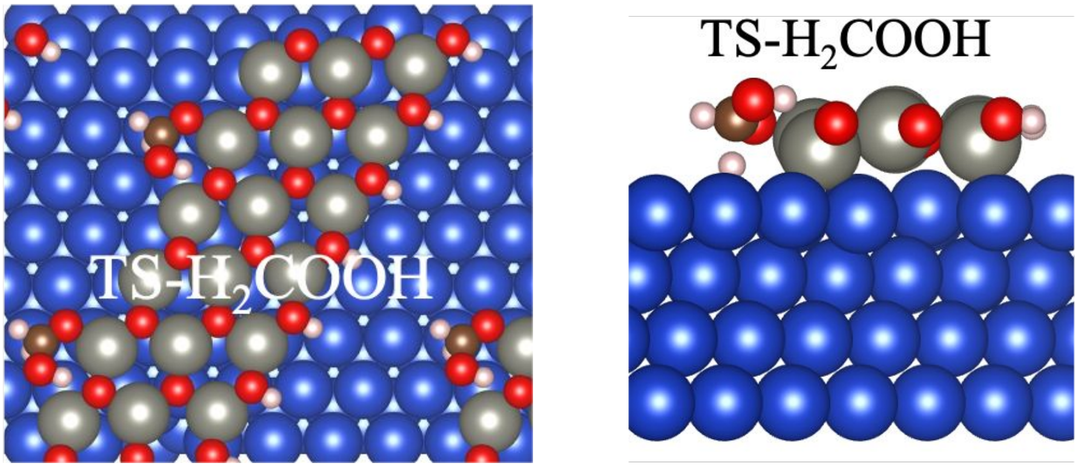

Fig. S12 Geometry of H_2COOH transition state at Interface B of 3x3-ZnO/7x4-Cu(111) model system (top and side view)

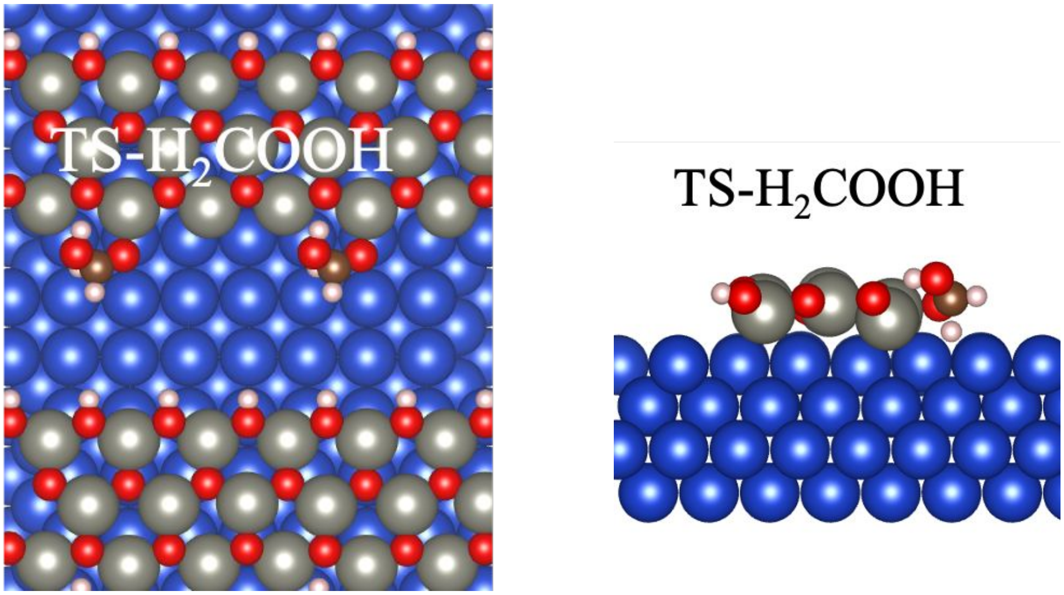

Fig. S13 Geometry of H_2COOH transition state at Interface B of 3x3-ZnO/4x6-Cu(100) model system (top and side view)

DRIFTS

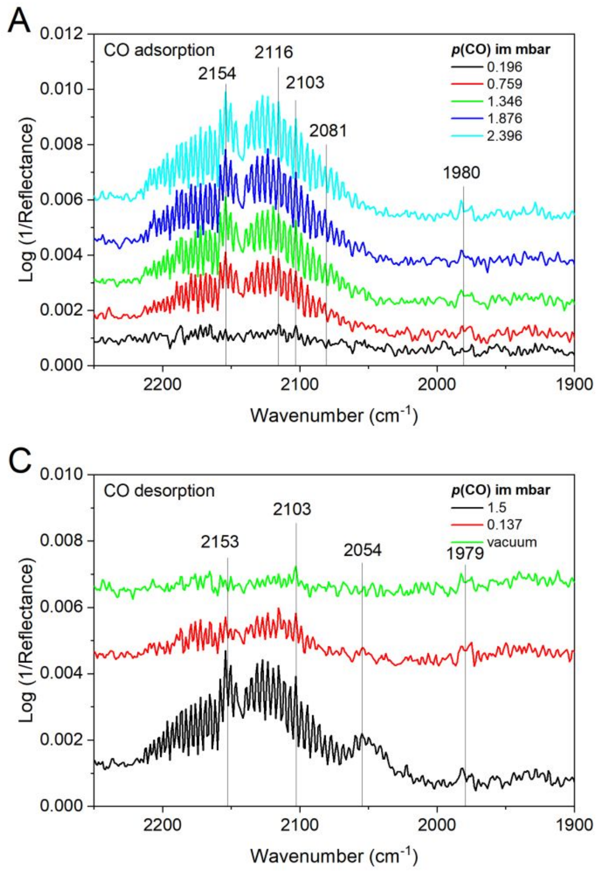

Fig. S14 DRIFTS spectra during CO adsorption/desorption measurements at -187°C. Spectra are shown for the Cu NPs on ZnO (A, C) and Cu NCs on ZnO (B, D).
